# The effectiveness of diabetes self-management education intervention on glycaemic control and cardiometabolic risk in adults with type 2 diabetes in low- and middle-income countries: A systematic review and meta-analysis

**DOI:** 10.1371/journal.pone.0297328

**Published:** 2024-02-02

**Authors:** Hasina Akhter Chowdhury, Cheryce L. Harrison, Bodrun Naher Siddiquea, Sanuki Tissera, Afsana Afroz, Liaquat Ali, Anju E. Joham, Baki Billah

**Affiliations:** 1 Department of Epidemiology and Preventive Medicine, School of Public Health and Preventive Medicine, Monash University, Melbourne, Australia; 2 Centre for Injury Prevention and Research, Bangladesh (CIPRB), Dhaka, Bangladesh; 3 Monash Centre for Health Research and Implementation–MCHRI, School of Public Health and Preventive Medicine, Monash University, Melbourne, Australia; 4 Department of Biochemistry and Pharmacology, Faculty of Medicine, Dentistry and Health Sciences, The University of Melbourne, Melbourne, Australia; 5 Pothikrit Institute of Health Studies (PIHS), Dhaka, Bangladesh; 6 Departments of Endocrinology and Diabetes, Monash Health, Melbourne, Australia; University of Benghazi, LIBYA

## Abstract

Diabetes mellitus (DM) poses a significant challenge to public health. Effective diabetes self-management education (DSME) interventions may play a pivotal role in the care of people with type 2 diabetes mellitus (T2DM) in low- and middle-income countries (LMICs). A specific up-to-date systematic review is needed to assess the effect of DSME interventions on glycaemic control, cardiometabolic risk, self-management behaviours, and psychosocial well-being among T2DM across LMICs. The MEDLINE, Embase, CINAHL, Global Health, and Cochrane databases were searched on 02 August 2022 and then updated on 10 November 2023 for published randomised controlled trials (RCTs) and quasi-experimental studies. The quality of the studies was assessed, and a random-effect model was used to estimate the pooled effect of diabetes DSME intervention. Heterogeneity (I^2^) was tested, and subgroup analyses were performed. Egger’s regression test and funnel plots were used to examine publication bias. The risk of bias of the included studies was assessed using the Cochrane risk-of-bias tool for randomized trial (RoB 2). The overall assessment of the evidence was evaluated using the Grading of Recommendations Assessment, Development, and Evaluation approach. A total of 5893 articles were retrieved, and 44 studies (n = 11838) from 21 LMICs met the inclusion criteria. Compared with standard care, pooled analysis showed that DSME effectively reduced the HbA1c level by 0.64% (95% CI: 0.45% to 0.83%) and 1.27% (95% CI: -0.63% to 3.17%) for RCTs and quasi-experimental design studies, respectively. Further, the findings showed an improvement in cardiometabolic risk reduction, diabetes self-management behaviours, and psychosocial well-being. This review suggests that ongoing support alongside individualised face-to-face intervention delivery is favourable for improving overall T2DM management in LMICs, with a special emphasis on countries in the lowest income group.

## Introduction

Diabetes mellitus (DM) is a prevalent public health concern [1], with an estimated 537 million (10.5%) adults aged between 20 to 79 affected globally in 2021 [2]. Among those adults, approximately 90% had type 2 diabetes (T2DM) [2, 3]. T2DM is the primary cause of major micro- and macro-vascular complications contributing to significant adverse clinical sequelae, including premature death [4]. In recent decades, the prevalence of T2DM has escalated more rapidly in low- and middle-income countries (LMICs) compared with high-income countries (HICs), with an estimated 79.4% of the global T2DM population residing in LMICs [2]. In 2021, the estimated global annual cost of diabetes treatment was 966 billion USD [2], imposing a substantial health and economic burden on individuals, their families, and healthcare systems [5–10].

The cornerstone of T2DM management is controlling glycosylated haemoglobin (HbA1c) and optimising cardiometabolic risk factors [11]. Self-management of healthy lifestyle strategies, typically involving optimisation of diet, increasing physical activity, and weight loss in those who are overweight and obese, are recommended as first-line interventions; however, these are highly dependent on individual health literacy, self-efficacy, and motivation [12]. For this reason, diabetes education is crucial in optimising self-management strategies by enhancing knowledge as well as by encouraging and consolidating behaviour-change skills [13, 14]. All of these can be addressed using diabetes self-management education (DSME) intervention [15–17]. DSME intervention includes educating patients through the application of self-care strategies (facilitating with the knowledge, skill and ability) in a cost-effective manner to enhance treatment adherence, diabetes self-management (diabetes knowledge and self-efficacy), lifestyle change (diet, physical activity and weight management where appropriate) and psychological well-being (health-related quality of life [HrQoL]) [15, 18, 19].

Previous systematic reviews and meta-analyses conducted in HICs demonstrate that DSME intervention is associated with improved glycaemic control, diabetes knowledge, self-efficacy, HrQoL [20–22], and reduction in all-cause mortality [23]. This includes a 0.4% reduction in HbA1c, a more than 5 mg/dl reduction in total cholesterol (TC) and a more than 1 mmol/L reduction in fasting blood glucose (FBG) when compared to standard care [24–29]. In addition, DSME intervention in HICs showed positive changes in diabetes-specific knowledge and lifestyle [30]. However, generalising evidence from HICs to LMICs needs to be interpreted with caution given cultural, ethnic, and economic disparities, as well as the variations among study populations [30, 31]. Recent reviews conducted in LMICs demonstrated that DSME intervention, short-term nutrition education and/or lifestyle modification intervention may enhance glycaemic control [30, 32–35] and anthropometric measures [33]. However, to our knowledge, limited attempts have been made in the literature to assess the effectiveness of DSME interventions on a comprehensive outcome measures in LMICs [36–39], which include the effectiveness in the change in diabetes control and cardiometabolic risk, diabetes self-management behaviours and psychosocial well-being. Thus, the aim of the present review is to comprehensively assess the effectiveness of DSME intervention on glycaemic control (eg. HbA1c/FBG), cardiometabolic risk factors (eg. WC, BMI, LDL, HDL, TC, TG, SBP, and DBP), diabetes self-management behaviours (eg. diabetes knowledge and self-care) and psychosocial well-being (eg. health-related quality of life) among people with T2DM living in LMICs and to explore intervention characteristics, as well as their mode of delivery, frequency, intensity and duration in relation to the improvement in outcomes.

## Methods

This systematic review and meta-analysis was registered with PROSPERO (CRD: 42022364447) and conducted according to the Preferred Reporting Items for Systematic Reviews and Meta-analyses (PRISMA) guidelines [40] (S1 Table).

### Selection criteria

#### Inclusion criteria

The Participant, Intervention, Comparison, Outcome and Study type (PICOS) framework (S2 Table) informed the inclusion and exclusion criteria. Participants included adults with T2DM residing in LMICs. Any form of educational intervention (e.g. self-management intervention with a variety of educational/behavioural components and/or lifestyle modification to diet and exercise) delivered in an LMIC to people with T2DM and targeting diabetes care management compared with standard care/usual care. Outcomes included any one or combination of the following: glycaemic control (HbA1c/fasting blood glucose [FBG]), cardiometabolic risk body mass index (BMI), waist circumference (WC), high-density lipoproteins (HDL), low-density lipoproteins (LDL), triglycerides (TG), total cholesterol (TC), systolic blood pressure (SBP), diastolic blood pressure (DBP), diabetes knowledge, self-efficacy and health-related quality of life (HrQoL). The study types included either RCT or quasi-experimental designs without language or time restrictions.

#### Exclusion criteria

Studies reporting on type 1 diabetes and gestational diabetes were excluded. Qualitative studies, editorials, commentary, reviews and case reports were excluded.

### Search strategy

Five electronic databases (MEDLINE, Embase, CINAHL, Global Health and Cochrane) were searched from their dates of inception through 02 August 2022 and updated on 10 November 2023 (S3 Table) by two authors (HAC and BNS) in consultation with a senior librarian at Monash University. A range of keywords relating to T2DM including educational intervention and model/tools of diabetes care were used, and the list of LMICs was based on the current World Bank Database [41].

### Study selection process

**Fig 1:**
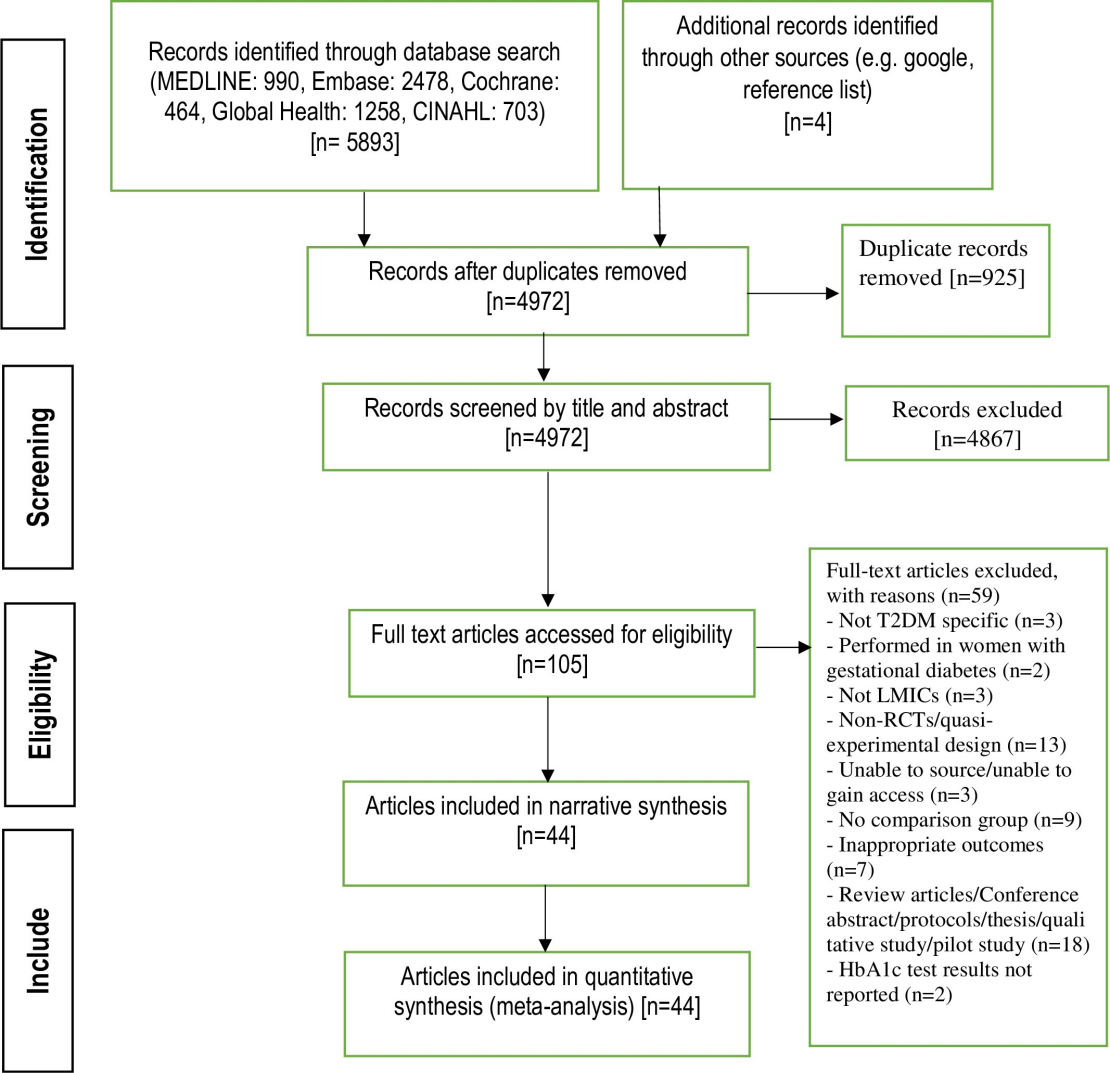
PRISMA flow diagram.

Retrieved articles were stored and managed using the citation software EndNote X20. Following the searches, two authors (HAC and BNS) independently screened all titles as well as abstracts and excluded studies that did not meet the inclusion criteria. A total of 105 articles were selected for a comprehensive full-text review. Following a review for accuracy, two authors (HAC, and BNS) independently reviewed the full text of these 105 articles, and any discrepancy was discussed with a third author (ST) with the supervision of senior author (BB). Finally, a set of 44 articles were selected to determine final article eligibility (Fig 1). A manual search of reference lists of included studies was also performed.

### Study outcomes

The primary outcome of this study was to assess any changes in glycaemic control (i.e. HbA1c or fasting blood glucose [FBG]) after intervention. Secondary outcomes were cardiometabolic risk factors (i.e. BMI, WC, HDL, LDL, TG, TC, SBP or DBP), HrQoL and changes in behavioural outcomes (i.e. diabetes knowledge and self-efficacy [S4 Table]).

### Data extraction

Data from the included articles were extracted independently by two authors (HAC and BNS) using Microsoft Excel. The following information was extracted: publication details (author/s, year of publication and journal), study characteristics (country, study design, setting, population and sample size), demographics (age of the participants), details of the intervention (type, frequency, intensity, intervention format, duration, number of educational sessions, intervention provider and mode of delivery of the intervention) as well as primary and secondary outcomes (i.e. HbA1c/FBG, BMI, WC, LDL, TG, TC, SBP, DBP, diabetes knowledge, self-efficacy and HrQoL). Discrepancies were discussed and resolved through consensus or arbitration between reviewers.

### Quality assessment

Study quality was appraised independently by two authors (HAC and BNS) using the revised Cochrane risk-of-bias tool for randomised trials (RoB 2) [42, 43] for randomised controlled trials, and the Joanna Briggs Institute (JBI) Critical Appraisal Checklist for quasi-experimental studies (non-randomised experimental studies) [44]. The Cochrane’s RoB 2 tool evaluates randomisation process, deviations from the intended interventions, missing outcome data, measurement of the outcome, and selection of the reported result [42]. For this review, the overall risk of bias was rated as high/low/some concerns, in agreement with the RoB 2 tool. Senior author (BB) was consulted to resolve instances of disagreement. A detailed description of the quality assessment has been provided as supporting information (S5 Fig and S6 Table).

### Assessment of certainty of the evidence

Grading of Recommendations, Assessment, Development, and Evaluations (GRADE) was used to evaluate the quality of the evidence [45]. GRADE pro-GDT was employed to summarise the quality of evidence [46]. The certainty of the evidence encompasses consideration of the within-study risk of bias which comprises methodological worth, indirectness of evidence, unexplained heterogeneity, imprecision and, probability of publication bias. The GRADE approach has following four levels of quality such as high-quality evidence that recommends that additional study is very unlikely to change our confidence in the estimate of effect size; moderate quality reflects further research as likely to have a vital impact on the estimate of effect size and may alter the estimate; low quality reveals that further research is very unlikely to have a significant influence on the current estimate of effect size and is likely to change the estimate; and very low quality suggests one is precise indeterminate about the estimate.

### Data analysis

All statistical analyses were performed using Stata V.16 (StataCorp, College Station, Texas, USA). A random-effects model was used to estimate pooled mean differences (MD) for HbA1c or FBG and other relevant quantitative data with a 95% confidence interval (CI). Heterogeneity was tested using the ꭓ2-test on Cochran’s Q statistic, which was calculated by means of H and I^2^ indices. I^2^ values of over 75% were considered to represent substantial heterogeneity [47]. Subgroup analyses were also performed with the covariates of income level of the country, intervention type, mode of delivery of the intervention and study quality to identify possible sources of heterogeneity. Egger’s regression test and funnel plots were used to examine publication bias [48]. As standard deviation of the mean change from baseline is defined as a common missing outcome data [49], and difficulties in running a meta-analysis without missing standard deviations (SDs). The following formula was used to calculate missing SDschange [50]:

SDchange = $\sqrt{\left({\mathrm{SD}}^{2}\;\mathrm{baseline}+{\mathrm{SD}}^{2}\;\mathrm{final})-(2*r*\mathrm{SD}\;\mathrm{baseline}*\mathrm{SD}\;\mathrm{final}\right)}$. If the SDbaseline and SDfinal values were known, the SDchange value was calculated by assigning a value of 0.7 to the r in the formula, to provide a conservative estimate as undertaken by previous systematic reviews [50]. All data are reported as a mean difference (95% confidence limits). Characteristics of the included studies are reported as mean (±SD) or number percentages as appropriate. In order to readability of the results, all p-values (where applicable) generated in the tables and forest plots have been approximated to three decimal places while reported in the results section. Statistical tests were considered significant at p-values ≤5% (≤0.05)

## Results

### Selection of studies

A total of 58974 articles were retrieved from the five databases (MEDLINE, Embase, Cochrane, global health and CINAHL) and manual searches. After removing duplicates through title and abstract screening, 105 articles were included for full-text review. Of those, 44 studies (n = 41 RCTs and n = 3 quasi-experimental studies) conducted in 21 LMICs that included 11,838 participants (5,887 in the intervention arm and 5,951 in the comparator arm) (Fig 1).

### Characteristics of the included studies

The characteristics of the included studies are reported in Table 1. Of the 44 studies, 21 were conducted in upper-middle-income countries [51–71], 21 in lower-middle-income countries [1, 38, 72–90], and two were conducted in low-income countries [91, 92], as grouped by the World Bank criteria [41]. The studies were conducted in diabetes clinics or hospitals (n = 15 [34%]), public or private hospitals/clinics (n = 21[48%]) and community settings/home-based locations (n = 8 [18%]). All community settings/home-based studies were conducted in the upper-middle-income countries except one from a low-income country [91]. No community-based studies were conducted in the Southeast Asian region. The HbA1c was reported most frequently (n = 42 [95%] studies), followed by FBG (n = 19 [43%]), BMI (n = 23 [52%]), WC (n = 10 [23%]), LDL (n = 18 [41%]), HDL (n = 17 [39%]), TC (n = 17 [39%]), TG (n = 12 [27%]), SBP (n = 20 [45%]), DBP (n = 17 [39%]), diabetes knowledge (n = 10 [23%]), self-efficacy (n = 7 [16%]), and HrQoL (n = 6 [14%]).

**Table 1:** Summary characteristics of the included studies.

Sl No	First author (year)	Study design	Country	Country by income	Sample size	Study duration (in weeks)	Age in years Mean (SD)	Mode of delivery of the intervention	Intervention format	Model/theory used	Intervention duration; number of sessions (min/session)	Type of intervention	Intervention provider	Settings	Outcome measures
1	Askari et al (2018) [72]	Randomised clinical trial	Iran	Lower middle income	108 (I: 54, C: 54)	12	I: 66.45 (3.40); C: 67.11 (3.25)	Face to face and telephone follow up	Group session	BASNEF model	12 weeks; 8 (70)	Lifestyle modification (focus on diet and exercise)	Researcher	Diabetes centre	HbA1c, FBS, TG, LDL, HDL
2	Azami et al (2018) [93]	Randomised control trial	Malaysia	Upper middle income	142 (I: 71, C: 71)	39	54.2 (11.8)	Face to face and telephone follow up	Group session	Nurse led DSME (diabetes self-management education)	12 weeks; 4 (120)	DSME intervention	Nurse	Urban primary and secondary outpatient endocrine clinic within a teaching hospital	HbA1c, TG, HDL, LDL, SBP, DBP, BMI, quality of life, self-efficacy
3	Baviskar et al (2021), [1]	Randomised control trial	India	Lower middle income	80 (I: 40, C: 40)	26	NR	Face to face	Group session	Self-care and diabetes realted educational intervention	24 weeks; NR	DSME intervention	Investigator and Medical Social worker	Malavni Urbran Health Training Centre	HbA1c, FBG, BMI, Quality of Life
4	Chow et al (2016) [51]	Non-clinical randomised controlled trial	Malaysia	Upper middle income	150 (I:75, C:75)	26	NR	Face to face and telephone reminder	Individual session	Home-based educational intervention	24 weeks; 2 (62)	DSME intervention	Pharmacist	Home based	HbA1c, diabetes knowledge
5	Debussche et al (2018) [91]	Randomised control trial	Mali	Low income	151 (I: 76 C: 75)	52	I: 53.9 (9.8); C: 51.1 (9.6)	Face to face	Group and individual session	Self-management educational intervention	52 weeks; 4 (120)	DSME intervention	Peer educators	Community	HbA1c, BMI, SBP, DBP, WC, diabetes Knowledge
6	Didarloo et al (2016) [73]	Randomised control trial	Iran	Lower middle income	90 (I: 45, C:45)	12	NR	Face to face	Group session	Collaborative and interactive teaching methods	12 weeks; 4 (60)	DSME intervention	Nurse	Diabetes clinic	HbA1c, Quality of Life
7	Ebrahimi et al (2016) [74]	Double blind Randomised clinical trial	Iran	Lower middle income	106 (I:53, C:53)	8	I: 46.97 (5.54); C:48.15 (6.52)	Face to face	Group session	Empowerment approach training	8 weeks; 5 to 7 (60 to 90)	DSME intervention	Nurse, endocrinologist and nutritionist	Diabetes center	HbA1c
8	Essien et al (2017) [75]	Individually-randomised controlled trial	Nigeria	Lower middle income	158 (I: 59, C:59)	26	All: 52.7; I:52.6; C: 52.8	Face to face and Mobile phone messages	Group session	Diet, nutrition and medication related education	24 weeks; 12 (120)	DSME intervention	Physician and nurse	Endocrinology clinic, Teaching Hospital	HbA1c
9	Gathu et al (2018) [76]	Non-blinded randomised clinical trial	Kenya	Lower middle income	140 (I:70, C:70)	26	All: 48.8 (9.8); (I: 50.2 (9.93); C: 47.5 (9.54)	Face to face and telephone reminders	Group session	Diabetes self-management education and support (DSMES): an empowerment and interactive teaching model	24 weeks; 6 (60)	DSME intervention	Family physician and diabetes educator	Family medicine clinic (private, urban-based) of a university hospital	HbA1c, SBP, DBP, BMI
10	Goldhaber-Fiebert et al (2003) [52]	Randomised conrol trial	Vietnam	Upper middle income	75 (I:40, C:35)	12	I: 60 (10); C: 57 (9)	Face to face	Group session	Community-based nutrition and exercise intervention	12 weeks; 11 (90)	Lifestyle modification (focus on diet and exercise)	Physician	Community centres	HbA1c, FBG, BMI, SBP, DBP, TC, HDLc, LDLc, TG
11	Goodarzi et al (2012) [77]	Randomised conrol trial	Iran	Lower middle income	100 (I:50, C:50)	12	I: 50.98 (10.32); C: 56.71 (9.77)	Text message	Individual session	Distance education via mobile phone text messaging	12 weeks; 48 (messages)	DSME intervention	Researcher	Hospital	HBA1c, TC, HDL, LDLc, TG, Knowledge, self-efficacy
12	Grillo et al (2016) [53]	Single-center, parallel-group, randomised study	Brazil	Upper middle income	131 (I: 69, C:62)	54	I: 61.7 (9.9); C: 63.2 (9.7)	Face to face	Group session	Education on diabetes care	7 weeks; 7 (120)	DSME intervention	Nurse	Primary care unit	HbA1c, BMI, WC, SBP, DBP, TC, LDL, HDL, TG
13	Hosseini et al (2017) [78]	Randomised control trial	Iran	Lower middle income	106 (I:53, C: 53)	26	I: 51.55 (8.3); C: 58.09 (1.6)	Face to face	Group session	PRECEDE model	4 weeks; 4 (120)	DSME intervention	General physician and specialist in health education and promotion	Diabetes clinic	HbA1c, BMI
14	Huo et al (2019) [55]	Randomised clinical trial	China	Upper middle income	502 (I: 251, C: 251)	26	All: 59.5	Text message	Individual session	A text messaging–based secondary prevention program with the regular automatic delivery of text messages.	26 weeks; 156 (text messages)	DSME intervention	Text messages	Hospital	HbA1c, FBG, SBP, LDL, BMI
15	Jain et al (2018) [79]	Open-label randomised controlled trial	India	Lower middle income	299 (I: 153, C:146)	24	I: 55.69 (10.94); C:57.42 (10.95)	Face to face and telephone reminder	Individual session	Combining face-to-face interaction with telephonic reminders by community health workers	24 weeks; 4 (home visits)	DSME intervention	Community health workers	Tertiary teaching institute	HbA1c, FBS, SBP, DBP, BMI, WC, TC, TG, LDLC, HDL
16	Jayasuria et al, (2015) [80]	Randomised control trial	Sri Lanka	Lower middle income	87 (I: 43, C: 42)	26	All: 51.4 (7.2)	Face to face	Group and individual session	Diabetes Self-Management-Sri Lanka (DSM-SL) model	26 weeks; 9 (60)	Lifestyle modification (diet and exercise)	Physician and nurse	Colombo North Teaching Hospital	HbA1c, SBP, TC, LDL, HDL, BMI, self-efficacy
17	Jiang et al (2019) [56]	Multicentre randomised controlled trial	China	Upper middle income	265 (I: 133, C: 132)	26	All: 56.91 (10.05)	Face to face	Group session	Structured education programme Self-Efficacy for Diabetes (C-SED) Diabetes Distress Scale (C-DDS) Summary of Diabetes Self Care Activities (C-SDSCA)	26 weeks; 4 (60 to 90)	DSME intervention	Physician and nurse	Multicentre at Bejing, Fujiam, Jiangxi	HbA1c, WC, BMI, blood pressure, TC, TG, LDL, HDL, diabetes knowledge, self-efficacy
18	Ju et al (2018) [57]	Cluster randomised control trial	China	Upper middle income	400 (I:200, C:200)	52	I: 67.8 (7.4); C: 68.8 (8)	Face to face	Group session	A community based peer support programe	52 weeks; 12 (120)	DSME intervention	Peer support/Leaders	Eight community health centres	HBA1c, FBG
19	Kong et al (2019) [58]	Group Randomized Experimental Study	China	Upper middle income	278 (I: 142, C: 136)	39	I: 69.12 (10.54); C: 71.48 (8.79)	Face to face	Group session	Chronic Care Model	39 weeks; 9 (NR)	DSME intervention	Physician, health manager and public health assistant	Community health service center	HbA1c, SBP, DBP, BMI, TC, LDL, HDL
20	Lamptey et al (2023) [38]	Single-blind randomised parallel comparator controlled multi-centre trial	Ghana	Lower middle income	206 (I:103; C:103)	13	I: 59; C: 57	Face to face	Group session	DESMOND: EXTENDing availability of self-management structured education programmes	13 weeks; 1 (720)	DSME intervention	Educator	Hospitals	HbA1c, WC, SBP, DBP, PAID
21	Li et al (2016) [59]	Randomized controlled trial	China	Upper middle income	196 (I: 98, C: 98)	4	I: 59.1 (4.6); C: 58.3 (4.1)	Face to face	Group session	Structured diet and/or exercise program (SDEP)	4 weeks; NR (NR)	DSME intervention	Health educators, doctors, and nutritionists	Hospital	HbA1c, FPG, BMI, TG, TC, HDL, LDL
22	Lou et al (2020) [60]	Randomised control trial	China	Upper middle income	1095 (I: 563, C: 532)	104	66.5 (8.7)	Face to face	Group session	Clinic-based intensified diabetes management model (C-IDM)	GPs and nurses: 24 weeks; NR (NR) Patients with diabetes: 78 weeks; 18 (NR)	DSME intervention	Not stated	Disease control centers, general hospitals and local clinics	HbA1c, FBG, SBP, DBP, BMI, TG, TC, HDL, LDL
23	Mohammadi et al (2018) [81]	A matched-pair design randomized controlled trial	Iran	Lower middle income	240 (I: 120, C: 120)	48	I: 51.2 (6.2); C: 51.4 (6.1)	Face to face	Group session	Health Belief Model (HBM)	12 weeks; 8 (120)	DSME intervention	Not stated	Golestan Hospital outpatient diabetes clinic	HbA1c, FBS, BMI, TC, TG, LDL, HDL, nutrition knowledge, quality of life, self-efficacy
24	Muchiri et al (2016) [61]	Randomised control trial	South Africa	Upper middle income	82 (I: 41, C: 41)	52	I: 59·4 (6.9); C: 58·2 (8.0)	Face to face	Group session	Nutrition education	52 weeks; 8 (120 to 180) and follow-up 6 (90)	DSME intervention	Health professionals	Community health centres	HbA1c, FBS, BMI, TC, TG, LDL, HDL
25	Myers et al (2017) [82]	Cluster randomised control trial	India	Lower middle income	239 (I: 85, C: 154)	52	46.3 (9.5)	Face to face	Group session	Nutrition practice guidelines	24 weeks; NR (NR)	Lifestyle modification (focus on diet)	Dietitian	Diabetes centres hospitals	HbA1c, BMI, TC, LDL, HDL, TG
26	Mash et al (2014) [62]	Pragmatic clustered randomized controlled trial	South Africa	Upper middle income	1570 (I: 710, C: 860)	52	I: 55.8 (11.5); C: 56.4 (11.6)	Face to face	Group session	Diabetes education programme	30 weeks; 4 (60)	DSME intervention	Educator	Community health centres	HbA1c, SBP, DBP, WC, TC, self-efficacy
27	Ojieabu et al (2017) [83]	Randomised control trial	Nigeria	Lower middle income	150 (I:75, C:75)	17		Face to face	Group session	Intervention of medication and treatment adherence	17 weeks; 4 (NR)	DSME intervention	Pharmacist	Endocrinology Clinic, Teaching Hospital	FBS, BMI, SBP, DBP
28	Ramadas et al (2018) [63]	Multi-centre randomised control trial	Malaysia	Upper middle income	128 (I: 66, C: 62)	104	I:49.6 (10.7); C:51.5 (10.3)	Web based	Web session	Malaysian Dietary Intervention for People with Type 2 Diabetes: An e-Approach (myDIDeA)	26 weeks; 12 (12)	Lifestyle modification (focus on diet)	Nutritionist	Public hospital	HbA1c, FBG, diabetes knowledge
29	Ramli et al (2016) [64]	Pragmatic cluster randomised controlled trial	Malaysia	Upper middle income	888 (I: 471, C:417)	104	I: 58 (0.48); C: 57 (0.5)	Face to face	Group session	EMPOWER-PAR (Participatory action research) interventions	52 weeks; 2 (NR)	DSME intervention	Physician, nurse, pharmacist and dietitian/nutritionist	Public primary care clinics	HbA1c, BMI, SBP, DBP, WC, TC, TG, LDL, HDL
30	Samtia et al (2013) [84]	Randomized study	Pakistan	Lower middle income	344 (I: 174, C: 170)	20	I: 46.1; C: 42.3	Face to face	Group session	Intervention regarding disease knowledge and self-care	20 weeks; NR (NR)	DSME intervention	Physician and pharmacist	Diabetes clinic at hospital	HbA1c, FBS, BMI
31	Sanaeinasab et al (2021) [85]	Randomised controlled trial	Iran	Lower middle income	80 (I: 40, C: 40)	30	All: 50.7 (5.9)	Face to face	Group session	Comprehensive systematic health education and promotion (SHEP) model	7 weeks; 6 (90)	DSME intervention	Not stated	Diabetic clinics	HBA1c, FBG, BMI, SBP, DBP, TC, HDL, LDL, TG
32	Salahshouri (2018) [86]	Randomised control trial	Iran	Lower middle income	145 (I: 73; C: 72)	26	I: 55.93 (12.4); C: 54.53 (9.43)	Face to face	Group session	Intervention based on psychological factors and nutrition	NR weeks; 8 (60)	Lifestyle modification (focus on diet)	Internal specialists, dietitians, diabetes experts, a psychologist, as well as a religious expert	Diabetic clinics and healthcare centres	HbA1c, FBS, self-efficacy
33	Tan et al (2011) [65]	Single-blind randomised control trial	Malaysia	Upper middle income	164 (I:82, C:82)	12	I: 54 (9.94); C:54 (10.74)	Face to face and telephone follow up	Group session	Self-efficacy theory	12 weeks; 3 (45)	DSME intervention	Not stated	Govt state hospital	HbA1c, diabetes knowledge, self-efficacy
34	Thanh et al (2021) [87]	Randomized controlled single-center trial	Vietnam	Lower middle income	364 (I: 182, C: 182)	52	All: 62.2 (9.3)	Face to face	Group session	Education on diet, exercise, drug therapy and adherence	12 weeks; 3 (45)	DSME intervention	Medical staff educators	Diabetes clinic	HbA1c, FBG, SBP
35	Wattana et al (2007) [66]	Randomised controlled trial	Thailand	Upper middle income	147 (I:75, C:72)	26	I: 58.40 (10.05); C: 55.14 (10.22)	Face to face	Group and individual session	Diabetes self-efficacy and diabetes self-management program	24 weeks; 5 (90 to 120) and one-off 2 home visits (45)	DSME intervention	Physician and researcher	Diabetic clinics	HbA1c, HrQol
36	Whittemore et al (2020) [67]	Randomised control trial	Mexico	Upper middle income	47 (I: 26, C: 21)	52	55.35 (8.75)	Face to face and follow up by phone calls	Group session	Si Yo Puedo DSME program	52 weeks; 7 (NR) and phone call every 2 weeks and text/picture messages sent daily for 6 months	DSME intervention	Nurse and social worker	Seguro Popular clinics	HbA1c, BMI, SBP, DBP, self-efficacy
37	Wichit et al (2017) [68]	Randomised controlled trial	Thailand	Upper middle income	140 (I:70, C:70)	13	I: 61.3 (11.6); C: 55.5 (10.5)	Face to face, home visit and telephone follow up	Group session	Self-efficacy theory	9 weeks; 3 (120)	DSME intervention	Nurse	Hospital	HbA1c, diabetes knowledge, HrQoL
38	Yan et al (2014) [92]	Randomised study	Mozambique	Low income	41(I: 31, C: 10)	12	I: 53 (2); C: 55 (3)	Face to face	Group session	Exercise training intervention	12 weeks; 36 to 60 (45)	Lifestyle modification (focus on exercise)	Not stated	Diabetes clinic	HbA1c, BMI, WC, SBP, DBP
39	Zhang et al (2018) [69]	Randomised study	China	Upper middle income	998(I:498, C: 500)	348	I: 50.8 (14.3); C: 52.6 (13.2)	Face to face	Group and individual session	Intervention on nutrition therapy, individualized exercise program, screening of complications	104 weeks; 24 (NR)	DSME intervention	Physician	Hospital	HbA1c, BMI, SBP, DBP, TC, HDL, LDL
40	Zheng et al (2019) [70]	Randomised controlled trial	China	Upper middle income	60 (I: 30, C:30)	104	52.22 (11.32)	Face to face	Group session	Diabetes self-management education programme	104 weeks; 2 (45)	DSME intervention	Therapist guidance	Hospital	HbA1c, FBG
41	Zhong et al (2015) [71]	Randomised study	China	Upper middle income	726 (I: 365; C: 361)	64		Face to face	Group session	Peer leader–support program for diabetes management	24 weeks; 12 (120)	DSME intervention	Peer leaders and staff of Community Health Service Centers (CHSCs)	Community	FBS, BMI, SBP, DBP, diabetes knowledge, self-efficacy
42	Al-Halaweh et al (2019) [88]	Quasi-experimental study	Palestine	Lower middle income	200 (I: 100; C: 100)	52	I: 56.58 (8.76); C: 57.9 (7.79)	Face to face	Group and individual session	Diabetes comprehensive care model (DCCM)	52 weeks; 4 (NR)	DSME intervention	Team of internal specialists, dietitians, diabetes experts, psychologist, and religious expert	Mobile diabetes clinic	Wt, Ht, BP, HbA1c, TC, Creatinine, Microalbuminuria
43	Pamungkas et al (2020) [89]	Quasi-experimental research	Indonesia	Lower middle income	60 (I: 30; C:30)	12	I: 56.5 (7.63); C: 54.2 (9.20)	Face to face	Group session	The diabetes mellitus self-management (DMSM) based coaching program	12 weeks; 3 (NR) and 1 (home visit)	DSME intervention	Researcher	Public health centers	HbA1c, SBP, DBP, BMI, TC, HDL, LDL
44	Kumari et al (2018) [90]	Quasi-experimental prospetive trial	India	Lower middle income	202 (I:102; C: 100)	65	I: 51.9 (9.3); C: 54 (8.6)	Face to face	Group and individual session	Lifestyle intervention holistic model (LIHM)	52 weeks; 6 (10 to 15)	Lifestyle modification (focus on diet)	Dietician, diabetes educator, physical trainer and diabetologist	Delhi Diabetes Research Centre	HbA1c, blood sugar fasting, blood sugar postprandial

The sample size in the studies ranged from 41 [92] to 1,570 [62], and the average age of the participants was 55 (SD: 6, range 42 to 71 years). The intervention durations ranged from four [59] to 348 weeks [69], with two-thirds (66.6%) of the studies lasting six months in duration. Standard care/usual care comprised the current standard of care as defined by the local programme or setting.

### Intervention characteristics

Overall, the majority of interventions utilised a behaviour-change approach focused on building knowledge, self-efficacy and self-management skills through counselling, coaching, brainstorming or supporting the control of T2DM and its related complications [S5 Table]. Five trials used DM self-management-based coaching programmes [54, 67, 80, 89, 91], four trials used the empowerment approach and interactive teaching model [63, 64, 74, 76], and three used the theory of self-efficacy as a theory or model to underpin intervention content [65, 66, 68]. Each of the following models was used by one trial only: the beliefs, attitudes, subjective norms and enabling factors (BASNEF) model [72]; the predisposing, reinforcing and enabling constructs in educational diagnosis and evaluation (PRECEDE) model [78]; the chronic care model [58]; clinic-based intensified diabetes management model (C-IDM) [60]; the health-belief model [81]; the comprehensive systematic health education and promotion (SHEP) model [85]; the diabetes comprehensive care model (DCCM) [88]; the structured DSME model [38] and the lifestyle intervention holistic model (LIHM) [90]. The remaining 23 trials [1, 51–53, 55–57, 59, 61, 62, 69–71, 73, 75, 77, 79, 82–84, 86, 87, 92] cited no theoretical framework or model used to inform the intervention designs.

Approximately 73% (n = 32) of the interventions were delivered using a face-to-face format, 20% (n = 9) utilising face-to-face intervention with telephone follow-up and 7% (n = 3) using a remotely delivered text message/web-based intervention. Intervention was delivered by healthcare professionals (e.g. physician, nurse, pharmacist, health educator, dietitian or nutritionist) in 32 trials [1, 38, 51–54, 56, 58, 59, 61–64, 66–70, 73–76, 78–80, 82–84, 86–88, 90], by the research team in three trials [72, 77, 89], by peer leaders or lay facilitators in three trials [57, 71, 91] and by trained educators in one trial [62]. Five trials did not report the type of intervention facilitator [60, 65, 81, 85, 92]. The intervention formats included groups (n = 33 [75%]), individuals (n = 4 [9%]), a combination of groups and individuals (n = 6 [14%]) and web-based (n = 1 [2%]) intervention strategies.

### Effect of DSME intervention on HbA1c and FBG control

**Fig 2:**
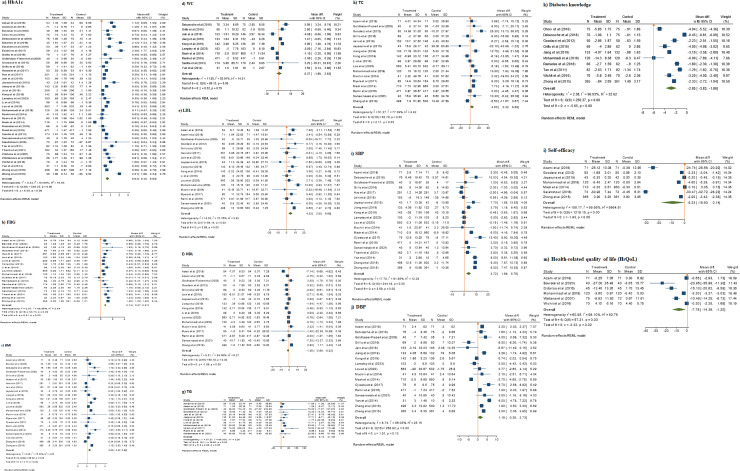
Meta-analysis results showing the effect of DSME interventions on clinical outcomes (a) HBA1c (b) FBG (c) BMI (d) WC (e) LDL (f) HDL (g) TG (h) TC (i) SBP (j) DBP, (k) diabetes knowledge, (l) self-efficacy, and (m) health-related quality of life of RCTs studies [Data are reported as mean difference (95% confidence limits)].

Of 41 RCT studies, 39 reported HbA1c (n = 10,500 participants). Upon meta-analysis, intervention significantly lowered HbA1c levels compared to the control, with a MD of 0.64% (95% CI: 0.64% to 0.83%; p = 0.001). Heterogeneity was very high between the studies (I^2^ = 94%) with no publication bias (Egger’s regression test, p = 0.068) (Fig 2 and Table 2).

**Table 2:** Summary results.

Study design	Outcome types	Measures	n	Mean change difference (with 95% CI), p-value	Effect of intervention	Heterogeneity (I^2^ in %)	Publication bias (Egger’s regression test p)
**RCTs**	Clinical	HbA1c	39	0.64 (0.45, 0.83), 0.001	Effective	94	0.0680
		FBG	19	0.74 (0.57, 0.91), 0.001	Effective	5996	0.5927
	Metabolic risk factors	BMI	23	0.60 (0.32, 0.88), 0.001	Effective	75	0.1738
		WC	10	0.37 (-1.89, 2.63), 0.001	Effective	93.01	0.6884
		LDL	18	4.33 (2.33–6.65), 0.001	Effective	71	0.0758
		HDL*	17	-1.35 (-2.69, 0.02), 0.05	Effective	84.06	0.2715
		TC	17	4.50 (0.32, 8.68), 0.03	Effective	779	0.5804
		TG	12	14.80 (8.18, 21.43), 0.001	Effective	69	0.0535
		SBP	20	3.72 (1.69, 5.75), 0.001	Effective	92	0.8676
		DBP	17	1.19 (-0.35, 2.73), 0.13	Effective	96	0.5148
	Diabetes self-managemnt behaviours	Diabetes knowledge*	10	-2.85 (-3.83, -1.79), 0.001	Effective	97	0.0070
		Self-efficacy*	7	-9.23 (-18.60, 0.14), 0.001	Effective	99	0.0001
	Psychosocial	HrQoL*	6	-7.78 (-14.36, -1.20), 0.02	Effective	98	0.0005
**Quasi-experimental design study**	Clinical	HbA1c	3	1.27 (-0.63, 3.17), 0.19	Effective	97	0.4515

*Negative results consider the positive effect of the intervention

Among 19 studies (n = 5,370 patients) that reported FBG, an overall decrease by 0.74 mmol/L (95% CI: 0.57% to 0.91%; p < 0.001) was observed in the intervention as compared with the control, with moderate heterogeneity (I^2^ = 59%) and no publication bias (Egger’s regression test, p = 0.592) (Table 2).

**Fig 3:**
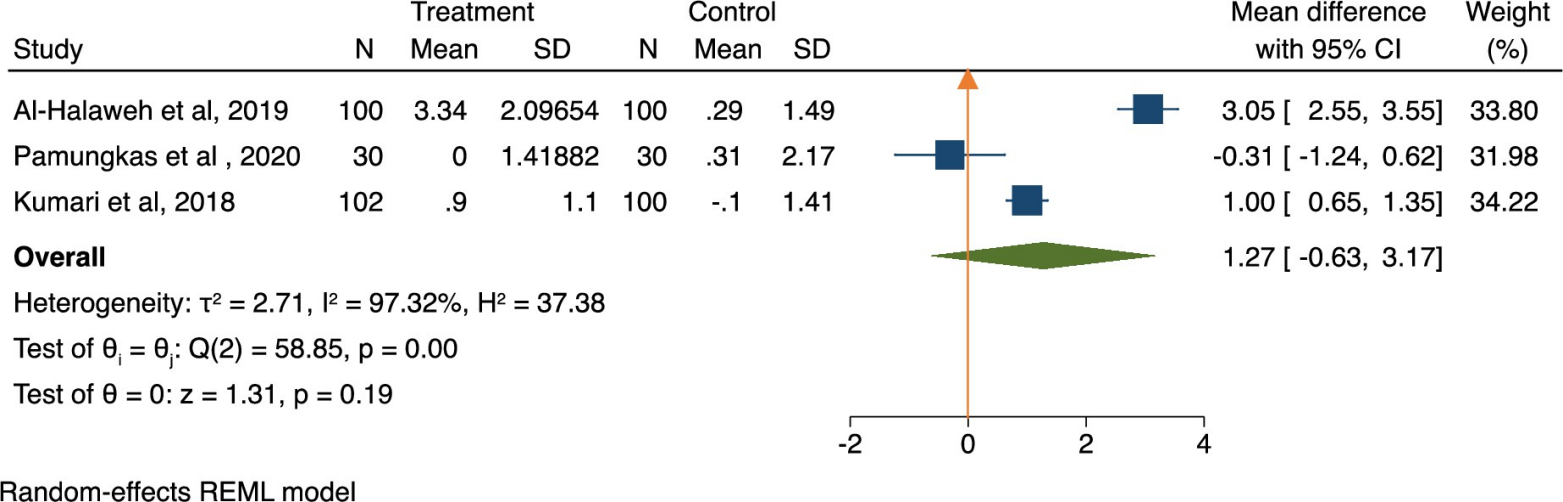
Meta-analysis results showing the effect of DSME interventions on glycaemic control (HbA1c) of quasi-experimental studies.

In trials with quasi-experimental designs, the findings showed a mean reduction in HbA1c of 1.27% (95% CI: -0.63% to 3.17%; p = 0.19) in the intervention as compared to the control (Fig 3). The I^2^ indicator was 97%, indicating a high heterogeneity with no publication bias (Egger’s regression test, p = 0.451) (Table 2). These studies did not report FBG levels.

### Effect of DSME interventions on cardiometabolic risk factors

DSME intervention reduced BMI by 0.60 kg/m^2^ (95% CI: 0.32% to 0.88%; p = 0.001, I^2^ = 75.33%) in 23 studies comprising 7,253 participants (Fig 2). Similarly, the results presented in Table 2 and forest plots showed a positive intervention effect on all cardiometabolic risk factors: WC (n = 4,173, MD 0.37, 95% CI: -1.89% to 2.63%; p = 0.001, I^2^ = 93%), LDL (n = 5803, MD 4.33, 95% CI: 2.33% to 6.65%; p = 0.001, I^2^ = 71%), HDL (n = 5301, MD -1.35, 95% CI: -2.69% to -0.02%; p = 0.05, I^2^ = 84.06%), TG (n = 6763, MD 14.80. 95% CI: 8.18% to 21.43%; p < 0.001, I^2^ = 69%), TC (n = 6,763, MD 4.50, 95% CI: 0.32% to 8.68%; p = 0.03, I^2^ = 779%), SBP (n = 8,128 MD 3.93, 95% CI: 1.83% to 6.04%; p <0.001, I^2^ = 926%) and DBP (n = 7,177, MD 1.19, 95% CI: -0.35% to 2.73%; p = 0.13, I^2^ = 96%). Moderate-to-high heterogeneity was observed across all forest-plot analyses of cardiometabolic risk factors.

### The effect of DSME intervention on diabetes knowledge, self-efficacy and HrQoL

Ten studies (n = 2,195) that evaluated knowledge of diabetes showed an improvement by MD of -2.85 (95% CI: -3.83% to -1.86%; p<0.001, I^2^ = 97%) with presence of publication bias (Egger’s regression test, p = 0. 0.007) (Fig 2). Impact on self-efficacy was addressed in seven studies (n = 1,588), showing an increase by 9.23 (95% CI: -18.60% to 0.14%; p = 0.05, I^2^ = 99%) with presence of publication bias (Egger’s regression test, p = 0.0070) (Fig 2). Six trials (n = 839) that reported HrQoL showed improvement by -7.78 (95% CI: -14.36% to –1.20%; p = 0·02, I^2^ = 98%). Publication bias was present in these studies (Egger’s regression test, p = 0.0005) (Fig 2).

### Subgroup/Sensitivity analysis

Moderate-to-high heterogeneity was observed across the studies regarding primary as well as secondary outcomes. In order to identify the sources of heterogeneity, subgroup/sensitivity analysis was conducted for the DSME intervention by the income level of the country, intervention type, mode of delivery of intervention and quality of the studies. As outlined in S1 Fig, DSME intervention showed that lower-middle-income countries had improvement in HbA1c with a MD of 0.75% (95% CI: 0.45% to 1.06%; p<0.001, I^2^ = 92%). Further, lifestyle modification (i.e. diet and/or exercise) intervention showed a greater effect on HbA1c reduction (MD: 0.69%, 95% 0.22% to 1.16%; p<0.001, I^2^ = 78%) than DSME interventions (MD: 0.63%, 95% CI: 0.42 to 0.86; p<0.001, I^2^ = 95%) (Table 3 and S2 Fig). In addition, subgroup analysis by mode of delivery of intervention showed that face-to-face intervention with periodic telephone follow-up had the highest efficacy on HbA1c reduction (MD: 1.02%, 95% CI: 0.63% to 1.40%; p<0.001, I^2^ = 86%) followed by face-to-face intervention alone (MD: 0.56%, 95% CI:0.32% to 0.80%; p<0.001, I^2^ = 95%) and text message or web-based intervention (MD: 0.33%, 95% CI: 0.17% to 0.49%; p = 0.35, I^2^ = 0.00) (Table 3 and S3 Fig). The quality of the trials with some concerns showed (S4 Fig) reduction in HbA1c with a MD of 0.66% (95% CI: 0.41% to 0.90%, p<0.001, I^2^ = 93%) compared with trails rated as high or weak. The S1–S4 Figs present subgroup analyses for BMI and lipid profiles (LDL, HDL, TG and TC) by the income level of the country, intervention type, mode of delivery of the intervention and quality of the study. In studies from low-income countries (MD: 0.87, 95% CI: -0.48% to 2.22%; p = 0.05, I^2^ = 75%), DSME intervention (MD: 0.63, 95% CI: 0.31% to 0.94%; p<0.001, I^2^ = 78%), face-to-face intervention (MD: 0.71, 95% CI: 0.41% to 1.01%; p<0.001, I^2^ = 74%) and trials evaluated as high risk (MD: 0.68, 95% CI: 0.18% to 1.18%, p<0.001; I^2^ = 82%) showed a better BMI reduction. Further, studies conducted in lower-middle income countries presented an improvement in LDL (MD: 7.32%, CI: 3.50% to 11.15%; p = 0.05, I^2^ = 56%), HDL (MD: -3.12, 95% CI: -5.62% to -0.62%; p<0.001, I^2^ = 89%), TC (MD:8.72, 95% CI: 0.88% to 18.32%; p<0.001, I^2^ = 83%) and TG (MD: 21.73, 95% CI: 15.26% to 28.19%; p<0.19, I^2^ = 10.66%).

**Table 3:** Subgroup analysis, based on the income level of the country, intervention type, mode of delivery of the intervention, and quality of the studies.

Subgroup	HbA1c	BMI	LDL	HDL	TG	TC
**Income level of the country**						
Low income	MD: 0.62 (0.13–1.11), I^2^ 67%	MD: 0.87 (-0.48–2.22), I^2^ 75%	N/A	N/A	N/A	N/A
Lower middle income	MD: 0.75 (0.45–1.06), I^2^ 92%	MD: 0.69 (0.32–1.06), I^2^ 46%	MD: 7.32 (3.50–11.15), I^2^ 56%	MD: -3.12 (-5.62 –-0.62), I^2^ 88%	MD: 21.73 (15.26–28.19), I^2^ 10%	MD: 8.72 (-0.88–18.32), I^2^ 83%
Upper middle income	MD: 0.55 (0.28–0.83), I^2^ 94%	MD: 0.53 (0.10–0.96), I^2^ 83%	MD: 2.78 (0.20–6.65), I^2^ 71%	MD: -0.34 (-1.69–1.00), I^2^ 69	MD: 8.85 (8.21–9.48), I^2^ 0.00%	MD: 2.05 (-1.99–6.09), I^2^ 660%
**Intervention type**						
Lifestyle modifications (diet and/or exercise)	MD: 0.69 (0.22–1.16), I^2^ 78%	MD:0.35 (-0.03–0.74), I^2^ 0.00%	MD:1.63 (-5.58–8.84), I^2^ 716%	MD: -1.77 (-6.75–3.22), I^2^ 91%	MD:42.24 (-4.21–88.70), I^2^ 70%	MD: 0.11 (-17.99–18.22), I^2^ 78%
Self-management	MD: 0.63 (0.42–0.85), I^2^ 95%	MD:0.63 (0.31–0.94), I^2^ 78%	MD: 4.33 (2.00–6.65), I^2^ 71%	MD: -1.14 (-2.38–0.11), I^2^ 74%	MD:13.64 (6.52–20.77), I^2^ 69%	MD: 4.86 (0.38–9.35), I^2^ 77%
**Mode of delivery of the intervention**						
Face-to-face	MD: 0.55 (0.32–0.78), I^2^ 94%	MD: 0.71 (0.41–1.01), I^2^ 74%	MD: 3.77 (0.77–6.77), I^2^ 75%	MD: -0.50 (-1.68–0.68), I^2^ 76%	MD: 16.93 (8.19–25.68), I^2^ 74%	MD: 3.15 (-1.08–7.39), I^2^ 754%
Face-to-face and telephone follow up	MD: 1.02 (0.63–1.40), I^2^ 86%	MD: 0.03 (0.56–0.62), I^2^ 0.00%	MD: 6.79 (3.58–10.01), I^2^ 0.00%	MD: -4.18 (-7.46 - -0.89), I^2^ 70%	MD: 11.30 (-1.79–24.39), I^2^ 62%	MD: 5.44 (-1.62–12.51), I^2^ 0.00%
Text messages or web-based	MD: 0.33 (0.17–0.49), I^2^ 0.00%	MD: -0.20 (-0.65–0.25), I^2^ N/A*	MD: 3.87 (-5.51–13.25). I^2^ 708%	MD: -3.32 (-6.63–0.0.01), I^2^.%N/A*	MD: 15.22 (-15.33–45.77), I^2^.% N/A*	MD: 25.30 (13.73–36.87), I^2.%^ NA*
**Quality of the studies**						
High	MD: 0.60 (0.30–0.91), I^2^ 94%	MD: 0.68 (0.18–1.18), I^2^ 82%	MD: 5.40 (-2.26–8.55), I^2^ 60%	MD: -1.87 (-5.09–1.34), I^2^ 92%	MD:-2.36 (-10.13–5.42), I^2^ 71%*	MD: -2.36 (-10.13–5.42), I^2^ 71%
Some concerns	MD: 0.66 (0.41–0.90), I^2^ 94%	MD: 0.49 (0.19–0.78), I^2^ 75%	MD: 3.94 (0.79–7.09), I^2^ 71%	MD: -0.69(-1.31–0.07), I^2^ 84%	MD: 7.26 (3.00–11.52), I^2^ 70%	MD: 7.26 (-3.00–11.52), I^2^ 70%

*N/A = not applicable, as ≤ one study in analysis.

In addition, intervention focused on DSME intervention demonstrated the highest MDs in LDL and TC (LDL: MD 4.33, 95% CI: 2.00% to 6.65%; p<0.001, I^2^ 71; and TC: MD 4.86 95% CI: 0.38% to 9.35%; p<0.001, I^2^ 77%) (Table 3). Lifestyle modification intervention alone showed better efficacy in reducing HDL (MD: -1.77, 95% CI: -6.75% to 3.22%; p<0.001, I^2^ = 91%) and TG (MD 42.24, 95% CI: -4.21 to 88.70; p<0.001, I^2^ 70%) (Table 3). Furthermore, face-to-face intervention with periodic telephone follow-up showed the highest MDs in LDL (MD 6.79, 95% CI: 3.58% to 10.01%; p = 0.52, I^2^ = 0.00%) and HDL (MD -4.18, 95% CI: -7.46% to -0.89%; p = 0.03, I^2^ = 0.03%) (Table 3). However, face-to-face intervention alone was more effective at reducing TG (MD 16.93, 95% CI:8.19% to 25.68%; p<0.001, I^2^ = 73.96%) (Table 3). Trials classified as high risk of bias showed improvement in the lipid profile of LDL (MD 5.40, 95% CI: -2.26% to 8.55%; p<0.010, I^2^ = 59.58%), HDL (MD -1.87, 95% CI: -5.09% to 1.34%; p = 0.001, I^2^ = 92%) and TG (MD 7.26, 95% CI: 3.00% to 11.52%; p = 0.001, I^2^ = 77% (Table 3).

### Risk of bias in the included studies

**Fig 4:**
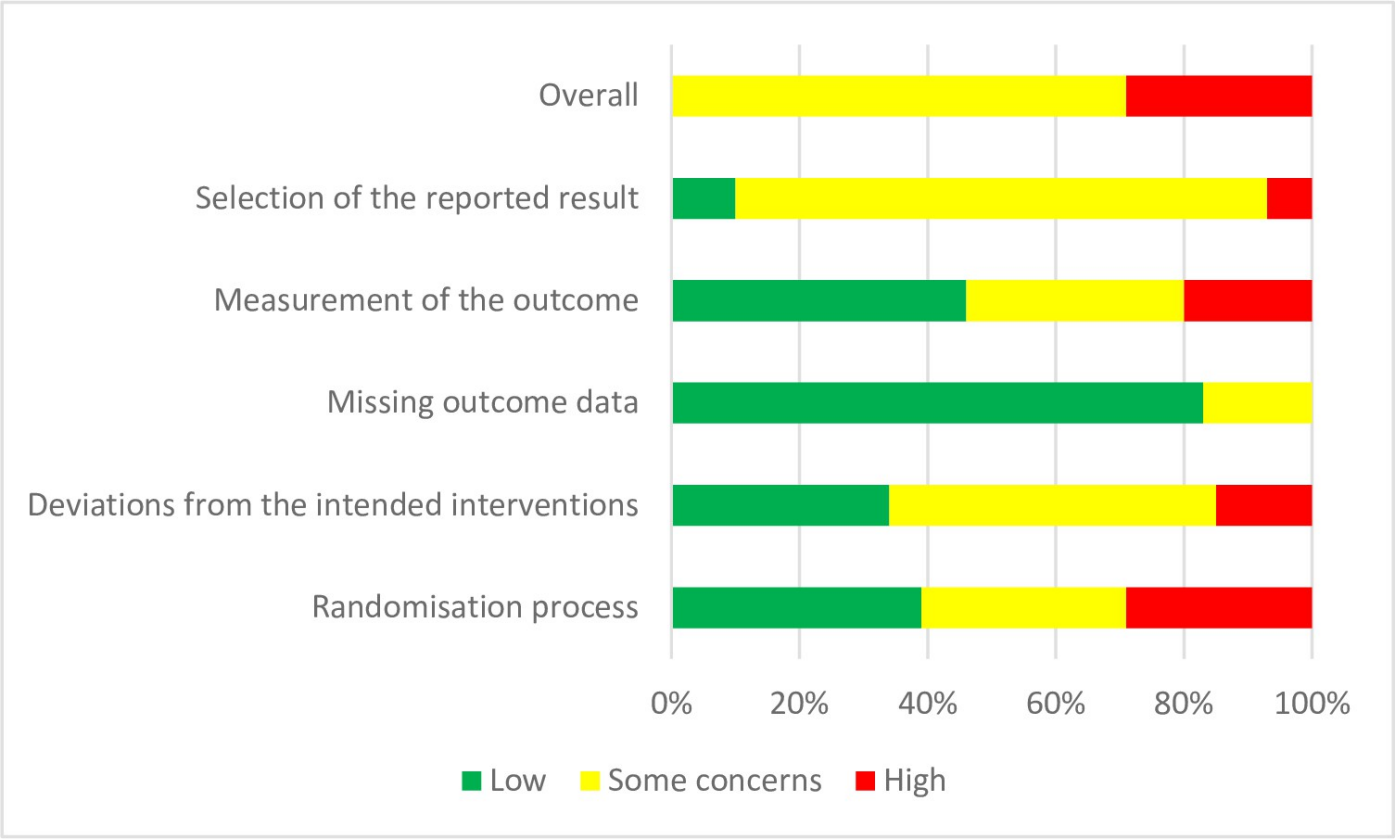
Risk of bias graph: Review authors’ judgements about each risk of bias item presented as percentages across all included studies.

The randomisation process for allocation was evaluated as low risk of bias in 16 studies [1, 30, 52–56, 61, 62, 65, 67, 68, 70, 73, 77, 85], and 13 studies measured as having some concerns of bias [51, 58–60, 63, 64, 75, 79–81, 84, 86, 87]. No trials were rated as low in all five components of the assessment tool. Deviations from the intended interventions were rated as high risk of bias in six studies [57, 69, 72, 82–84]. The risk of bias was rated as some concerns due to missing outcome data in seven studies [51, 59, 71, 76, 77, 85, 93]. Regarding measurement of the outcome reporting, eight studies [54, 69–72, 80, 81, 85, 92] were apparent as high risk of bias. However, for the selection of the reported results, four studies were evaluated as low risk of bias [53, 74, 86, 91], and three studies were assessed as high risk of bias [58, 75, 93]. The overall risk of bias for studies is summarised in Fig 4 and the risk of bias in individual study is reported in S5 Fig.

A quality assessment was carried out for each of the quasi-experimental studies using the JBI Critical Appraisal Checklist [44, 89, 90]. However, the assessment was a subjective measure that was dependent on the author carrying out the assessment. As per the appraisal checklist, three studies [88–90] were considered and included in the meta-analysis. The details are shown in S6 Table.

### Publication bias

The presence of publication bias for RCTs was visually assessed using a funnel plot for the primary outcome (HbA1c), which showed that there was no publication bias (Table 2). This was supported by the Egger’s test (p = 0.0680). Publication bias was also assessed for the secondary outcomes and presented in the Table 2, which showed that there was no publication bias for FBG (p = 0.5927), BMI (p = 0.1738), WC (p = 0. 6884), LDL (p = 0.0758), HDL (p = 0.2715), TC (p = 0.5804), TG (p = 0.0535), SBP (p = 0.8676) and DBP (p = 0.5148). Publication bias, however, was present for HrQoL (p = 0.0005), self-efficacy (p < 0.001) and diabetes knowledge (p = 0.0070). Regarding quasi-experimental studies, no publication bias was observed for HbA1c (p = 0.4515) (Table 2).

### Overall quality of the evidence

The GRADE approach was employed to assess the overall quality of evidence, and the results are summarized in the main comparison’s findings. Findings showed that the overall certainty of evidence for HDL and WC were moderate, which suggests further studies will increase our confidence in the estimate of effect size. The quality of the evidence for HbA1c, FBG, and BMI were low, which reflects that the effect size is limited and the true effect may be substantially different from the estimate of the effect size. The quality of evidence for LDL, TC and TG were very low, which showed that the true effect is probably markedly different from the estimated effect (S7 Table).

## Discussion

This systematic review and meta-analysis aimed to systematically examine the efficacy of DSME interventions on overall T2DM management and cardiometabolic outcomes. Pooled data were used covering 11,838 participants across 44 studies conducted in 21 LMICs. Comprehensive assessment was conducted to evaluate the effectiveness of DSME intervention on 13 outcomes measures including HbA1c control, cardiometabolic risk factors, self-efficacy, diabetes knowledge and psychosocial well-being factors among people with T2DM in LMICs. The outcomes were compared with those generated by standard care across both RCT and quasi-experimental trials. Consequently, a greater number of studies than the earlier reviews were included. This review and meta-analysis demonstrated that DSME intervention leads to better glycaemic control as compared to lifestyle modification intervention alone. Further, it also shows that face-to-face interventions followed by periodic phone calls results in better glycaemic control compared with only face-to-face or remote delivery strategies. The findings suggest that ongoing support is important in optimising intervention efficacy.

Compared with the standard care, this review showed that DSME intervention reduced HbA1c by 0.64% (95% CI: 0.45% to 0.83%) and 1.27% (95% CI: -0.63% to 3.17%) in RCTs and quasi-experimental design studies, respectively. This finding is consistent with previous reviews [20, 21, 93, 94] that reported a reduction in HbA1c levels by 0.83% (95% CI: 1.17% to 0.49%, n = 18 studies) [94] and 0.26% (95% CI: 0.05 to 0.48 n = 31 studies) [25] after DSME interventions. A decrease in HbA1c levels is known to reduce micro- and macro-vascular complications of people with T2DM in long-term follow-up [95–97]. Thus, DSME intervention should be a priority for optimising glycaemic control among people with T2DM in LMICs.

This review demonstrated that DSME intervention leads to significant improvement in FBG and other cardiometabolic risk factors (i.e. BMI, WC, SBP, DBP, LDL, HDL, TG and TC). The findings are in line with those of the previous review that showed the positive effects of group-based self-management education interventions on HbA1c, FBG, body weight, WC, TG and diabetes knowledge [98]. Another review, however, showed that there was no effect of community-based educational interventions on SBP and DBP [99]. Overall, these findings support the potential clinical, behavioural and psychological efficacy of DSME intervention in patients with T2DM.

Adults with diabetes or other metabolic diseases are more likely to have lower self-efficacy, knowledge about their illness and HrQoL [100] as compared with individuals without diabetes and metabolic syndrome. This meta-analysis showed that DSME intervention effectively increased self-efficacy, which is supported by a previous systematic review [101]. Additionally, in a tailored web-based intervention, patients with the highest self-efficacy had better outcomes; therefore, self-efficacy may play a moderating role in intervention outcomes and thus should be considered in tailoring DSME intervention for people with diabetes [102]. Peyrot and Rubin [103] found that those who had the worst self-care, improved the most following DSME intervention and that those with higher self-efficacy had a higher level of self-care behaviours. Self-efficacy provides the confidence necessary to overcoming disease barriers [104] and it receives the most consistent support as a strong determinant of diabetes self-care behaviours [105]. Further, in the present review, diabetes knowledge was significantly improved in the intervention group compared to controls (MD -2.85; 95% CI: -3.83% to -1.79%, p<0.001). Several meta-analyses have similarly shown that DSME interventions are associated with significant improvements in knowledge of T2DM [94, 106, 107]. Our results also showed that DSME intervention leads to improvement in HrQoL, as reported previously [108]. Other reviews have also demonstrated that DSME and behavioural modification improve HrQoL, which in turn impacts self-care and patients’ perceptions about diabetes care [109–112].

Subgroup analyses were performed by the income levels of the countries, intervention types, modes of delivery of the intervention, and quality of the studies. The analysis showed an overall improvement in HbA1c, BMI, LDL, HDL, TG and TC in the LMICs; however, low-income countries had a higher improvement in BMI (MD: 0.87, 95% CI: -0.48 to 2.22). It is possible that health-educational attainment has a direct impact on BMI. In addition, individuals with T2DM in low-income countries may be more physically active due to their need to secure income and also due to limited access to private transportation, leading to a less sedentary lifestyle as compared to those living in lower-middle-income countries [113]. In relation to intervention types, a noteworthy finding in this review is that people with T2DM who received DSME intervention had better BMI, LDL and TC reduction than those who received lifestyle (diet and physical activity) modification alone. This finding is similar to some [33, 34, 114] but not all [10] previous reviews reporting DSME intervention having a better effect on HbA1c control and BMI reduction. In addition to HbA1c and BMI, this current review demonstrated the efficacy of DSME interventions and lifestyle modification intervention in LDL, HDL, TG and TC. Another notable finding of this review is that the face-to-face interventions with periodic telephone follow-up results in better effects on glycaemic control and cardiometabolic risk than face-to-face or text message/web-based interventions alone, which is in line with the National Services Scheme by Diabetes Australia [115]. Periodic phone calls encouraging and reminding patients to practice self-management behaviours consistently over time improves their adherence to overall diabetes control [116]. Thus, face-to-face interventions with periodic telephone follow-up should be prioritised in future DSME intervention programmes for better T2DM management.

This systematic review and meta-analysis is noteworthy in terms of its synthesis of the evidence of outcomes through inclusion of trials using both RCTs and quasi-experimental intervention designs. Overall, it comprehensively summarises the potential clinical, behavioural and psychosocial efficacies of DSME interventions among people with T2DM in LMICs. In addition, five electronic databases were meticulously searched by the authors. As a result, a larger number of trials were identified leading to an impressive sample size of 11,838 participants. This review, however, has a few limitations. First, only a small number of studies were found from low-income countries. Second, the majority of the studies reported outcomes from less than one year follow-up, therefore the long-term effectiveness of DSME intervention in the management of T2DM population cannot be demonstrated. Third, high heterogeneity was observed in the meta-analyses for most of the outcome measures, which is likely due to variation in intervention programme design across the studies [99] as typically noted in intervention programmes of this nature. Fourth, no trial was categorised as low risk in all five components of the ROB 2 assessment tool. Particularly, randomisation process, deviations from the intended interventions, and measurement of the outcome were the most common risks of bias among the RCTs; hence, a prudent approach is warranted when interpreting the results of this present review. It is therefore recommended to follow the CONSORT statement [117] for parallel-group randomised trials to reduce the risk of biases when designing the methodology of the future RCTs. Further, the assessment of outcomes data was measured in heterogeneous ways in the included studies of this review and the certainty of evidence is not sufficient to assert the effectiveness of interventions among patients with T2DM. Hence, to enhance the certainty of evidence regarding the efficacy of these interventions, future RCTs should address the limitations observed in existing research in the literature.

## Conclusion

In conclusion, this systematic review and meta-analysis may have found a positive effect of DSME on the clinical and cardiometabolic risk factors, diabetes self-management behaviours and psychosocial well-being of people with T2DM in LMICs. Therefore, DSME interventions may enhance disease management and support to improve self-care strategies for people with T2DM. Further, interventions utilising a face-to-face delivery coupled with periodic ongoing support may be useful in improving glycaemic and lipid control as well as anthropometric measures. This study suggests that ongoing support alongside individualised face-to-face intervention delivery needs to be prioritised in order to improve overall T2DM management in LMICs, with a special emphasis on countries in the lowest income groups.

## Supporting information

S1 Table PRISMA checklist 2020. (DOCX)

S2 Table Eligibility criteria (PICOS). (DOCX)

S3 Table Search strategy. (DOCX)

S4 Table Primary and secondary outcomes. (DOCX)

S5 Table Other characteristics (intervention description) of the included studies. (DOCX)

S6 Table Risk of bias summary for quasi-experimental studies. (DOCX)

S7 Table GRADEpro level of quality evidence assessment. (DOCX)

S1 Fig Subgroup meta-analysis results showing the effect of interventions on (A) HbA1c, (B) BMI, (C) LDL, (D) HDL, (E) TG, and (F) TC based on the income level of the country.
(TIF)

S2 Fig Subgroup meta-analysis results showing the effect of interventions on (A) HbA1c, (B) BMI, (C) LDL, (D) HDL, (E) TG, and (F) TC based on intervention type.
(TIF)

S3 Fig Subgroup meta-analysis results showing the effect of interventions on (A) HbA1c, (B) BMI, (C) LDL, (D) HDL, (E) TG, and (F) TC based on the mode of delivery of intervention.
(TIF)

S4 Fig Subgroup meta-analysis results showing the effect of interventions on (A) HbA1c, (B) BMI, (C) LDL, (D) HDL, (E) TG, and (F) TC based on the quality of study.
(TIF)

S5 Fig Risk of bias summary (red, yellow, and green solid circle represents high risk of bias, some concerns risk of bias, and low risk of bias respectively): Review authors judgements about risk of bias item for each included study. (TIF)
