# Supplementary material for: The effectiveness of diabetes self-management education intervention on glycaemic control and cardiometabolic risk in adults with type 2 diabetes in low- and middle-income countries: A systematic review and meta-analysis
Source: PLoS One. 2024 Feb 2;19(2):e0297328. doi: 10.1371/journal.pone.0297328 (PMC10836683; doi:10.1371/journal.pone.0297328)
Supplement: S2 Table — (DOCX) [file pone.0297328.s002.docx]

**Table S2** Eligibility criteria (PICOS)

| **PICOS** | **Inclusion Criteria** | **Exclusion Criteria** |
| --- | --- | --- |
| Population | People with type 2 diabetes mellitus) (aged18 years and above) | Patients with other diseases |
| Intervention | Educational intervention for diabetes care management |  |
| Comparator | No intervention; comparison group with usual care/treatment (no restrictions on the definition on usual care/treatment) |  |
| Outcomes | Effect of the intervention on clinical, psychosocial and self-management | Other than clinical (e.g. economic outcomes) |
| Study Design | Randomised control trials and quasi-experimental design studies | Cross-sectional, book chapters, notes, comments, reviews, systematic literature reviews, meta-analyses, conference abstracts, editorials, case reports/series, animal or in-vitro studies |
| Time frame | Till 02 August 2022 and then updated 10 November 2023 |  |
| Geography | Low to middle-income countries |  |
| Language | English and non‐English |  |
