# Supplementary material for: The effectiveness of diabetes self-management education intervention on glycaemic control and cardiometabolic risk in adults with type 2 diabetes in low- and middle-income countries: A systematic review and meta-analysis
Source: PLoS One. 2024 Feb 2;19(2):e0297328. doi: 10.1371/journal.pone.0297328 (PMC10836683; doi:10.1371/journal.pone.0297328)
Supplement: S3 Table — (DOCX) [file pone.0297328.s003.docx]

**Table S3** Search strategy

| **#** | **Search History (**MEDLINE) |
| --- | --- |
| 1 | Diabetes Mellitus, Type 2/ |
| 2 | (diabet* adj1 (type 2 or type ii)).mp. |
| 3 | (T2DM or Tiidm or niddm).mp. |
| 4 | (noninsulin depend* or non-insulin depend* or mature-onset diabet* or adult-onset diabet*).mp. |
| 5 | 1 or 2 or 3 or 4 |
| 6 | education/ or education, distance/ or health education/ or consumer health information/ or health literacy/ or health promotion/ or patient education as topic/ |
| 7 | (education or learning).mp. |
| 8 | 6 or 7 |
| 9 | ((healthcare or care) adj model?).mp. |
| 10 | ("model* of care" or "model of healthcare").mp. |
| 11 | 9 or 10 |
| 12 | 5 and 11 |
| 13 | self care/ or self administration/ or self medication/ or self-testing/ or blood glucose self-monitoring/ |
| 14 | (self adj (care or monitor* or test* or medication* or administ* or manag* or treat* or help)).mp. |
| 15 | ((patient or self) adj (decision* or choice or determin*)).mp. |
| 16 | 13 or 14 or 15 |
| 17 | 5 and 16 |
| 18 | (model or models).mp. |
| 19 | 17 and 18 |
| 20 | 12 or 19 |
| 21 | Developing Countries/ |
| 22 | (indonesia* or malaysia* or thailand* or vietnam* or bangladesh* or india or nepal* or pakistan* or sri lanka*).mp. |
| 23 | ((developing or low* income or middle income) adj (countr* or nation?)).mp. |
| 24 | ((developing or third) adj world).mp. |
| 25 | 21 or 22 or 23 or 24 |
| 26 | 20 and 25 |
| 27 | (resource-limit* or resource-poor or low-resource* or limited-resource* or resource-constrain* or constrain*-resource* or under-resource* or poor*-resource* or resource-scarce* or scarce*-resource* or low-income or middle-income or lowincome or middleincome or (low adj3 middle-income)).mp. |
| 28 | ((developing or underdeveloped or under-developed or emerging or less-developed or least-developed or less-economically developed or least-economically developed or less-affluent or least-affluent or deprived or poor) adj (country or countries or nation or nations or region or regions or economy or economies)).mp. |
| 29 | ((developing or underdeveloped or under-developed or less-developed or least-developed) adj (population* or world)).mp. |
| 30 | (third-world* or thirdworld* or 3rd-world* or lmic or lmics or lami countr* or lalmi countr* or transitional countr*).mp. |
| 31 | (low* adj (gdp or gnp or gross domestic or gross national)).mp. |
| 32 | ((underserved or under served) adj (countr* or nation? or population*)).mp. |
| 33 | 25 or 27 or 28 or 29 or 30 or 31 or 32 |
| 34 | 20 and 33 |
| 35 | (Mumbai or Delhi or Bangalore or Hyderabad or Ahmedabad or Chennai or Kolkata or Surat or Pune or Jaipur or Lucknow or Kanpur or Nagpur or Indore or Thane or Bhopal or Visakhapatnam or Pimpri Chinchwad or Patna or Vadodara or Ghaziabad or Ludhiana or Agra or Nashik or Faridabad or Meerut or Rajkot or Kalyan Dombivli or Vasai Virar or Varanasi or Srinagar or Aurangabad or Dhanbad or Amritsar or Navi Mumbai or Allahabad or Howrah or Ranchi or Gwalior or Jabalpur or Coimbatore or Vijayawada or Jodhpur or Madurai or Raipur or Kota or Chandigarh or Guwahati or Solapur or Hubli Dharwad or Tiruchirappalli or Bareilly or Mysore or Tiruppur or Gurgaon or Aligarh or Jalandhar or Bhubaneswar or Salem or Mira Bhayandar or Warangal or Jalgaon or Guntur or Bhiwandi or Saharanpur or Gorakhpur or Bikaner or Amravati or Noida or Jamshedpur or Bhilai or Cuttack or Firozabad or Kochi or Nellore or Bhavnagar or Dehradun or Durgapur or Asansol or Rourkela or Nanded or Kolhapur or Ajmer or Akola or Gulbarga or Jamnagar or Ujjain or Loni or Siliguri or Jhansi or Ulhasnagar or Jammu or Sangli Miraj or Kupwad or Mangalore or Erode or Belgaum or Ambattur or Tirunelveli or Malegaon or Gaya or Thiruvananthapuram or Udaipur or Maheshtala or Davanagere or Kozhikode or Kurnool or Rajpur Sonarpur or Rajahmundry or Bokaro or South Dumdum or Bellary or Patiala or Gopalpur or Agartala or Bhagalpur or Muzaffarnagar or Bhatpara or Panihati or Latur or Dhule or Tirupati or Rohtak or Korba or Bhilwara or Berhampur or Muzaffarpur or Ahmednagar or Mathura or Kollam or Avadi or Kadapa or Kamarhati or Sambalpur or Bilaspur or Shahjahanpur or Satara or Bijapur or Kakinada or Rampur or Shimoga or Chandrapur or Junagadh or Thrissur or Alwar or Bardhaman or Kulti or Nizamabad or Parbhani or Tumkur or Khammam or Ozhukarai or Bihar Sharif or Panipat or Darbhanga or Bally or Aizawl or Dewas or Ichalkaranji or Karnal or Bathinda or Jalna or Eluru or Barasat or Kirari Suleman Nagar or Purnia or Satna or Mau or Sonipat or Farrukhabad or Sagar or Durg or Imphal or Ratlam or Hapur or Arrah or Anantapur or Karimnagar or Etawah or Ambarnath or North Dumdum or Bharatpur or Begusarai or New Delhi or Gandhidham or Baranagar or Tiruvottiyur or Pondicherry or Sikar or Thoothukudi or Rewa or Mirzapur or Raichur or Pali or Ramagundam or Haridwar or Vijayanagaram or Katihar or Nagercoil or Sri Ganganagar or Karawal Nagar or Mango or Thanjavur or Bulandshahr or Uluberia or Katni or Sambhal or Singrauli or Nadiad or Secunderabad or Naihati or Yamunanagar or Bidhannagar or Pallavaram or Bidar or Munger or Panchkula or Burhanpur or Raurkela or Kharagpur or Dindigul or Gandhinagar or Hospet or Nangloi Jat or Malda or Ongole or Deoghar or Chapra or Haldia or Khandwa or Nandyal or Morena or Amroha or Anand or Bhind or Bhalswa Jahangir Pur or Madhyamgram or Bhiwani or Berhampore or Ambala or Morbi or Fatehpur or Raebareli or Mahaboobnagar or Chittoor or Bhusawal or Orai or Bahraich or Vellore or Mehsana or Raiganj or Sirsa or Danapur or Serampore or Sultan Pur Majra or Guna or Jaunpur or Panvel or Shivpuri or Surendranagar or Dudhrej or Unnao or Chinsurah or Alappuzha or Kottayam or Machilipatnam or Shimla or Adoni or Udupi or Tenali or Proddatur or Saharsa or Hindupur or Sasaram or Hajipur or Bhimavaram or Kumbakonam or Dehri or Madanapalle or Siwan or Bettiah or Guntakal or Srikakulam or Motihari or Dharmavaram or Gudivada or Phagwara or Narasaraopet or Suryapet or Miryalaguda or Tadipatri or Karaikudi or Kishanganj or Jamalpur or Ballia or Kavali or Tadepalligudem or Amaravati or Buxar or Jehanabad or Aurangabad or Gangtok).mp. |
| 36 | (Sabaragamuwa or Uva province or Kandy or Trincomalee or Anuradhapura or Jaffna or Kurunegala or Ratnapura or Galle or Badulla or Colombo or Ampara or Batticaloa or Gampaha or Hambantota or Kalutara or Kegalle or Kilinochchi or Mannar or Matale or Matara or Monaragala or Mullaitivu or Nuwara Eliya or Polonnaruwa or Puttalam or Vavuniya or Dehiwala or Moratuwa or Sri Jayawardenapura or Kotte or Negombo or Kalmunai or Vavuniya or Katunayake or Dambulla or Kolonnawa or Anuradhapura).mp. |
| 37 | (Dhaka or Chittagongor Rajshahi or Khulna or Sylhet or Mymensingh or Barisal or Rangpur or Comilla or Narayanganj or Gazipur or Bogra or Kushtia or Jessore or Cox's Bazar or Manikganj or Brahmanbaria or Dinajpur or Nawabganj or Tangail or Sirajganj or Chandpur or Feni or Jamalpur or Naogaon or Narsingdi or Pabna or Maijdee or Faridpur or Tongi or Rangpur or Savar or Siddhirganj or Jessore).mp. |
| 38 | (Kathmandu or Pokhara or Lalitpur or Biratnagar or Birganj or Dharan or Bharatpur or Bhim Dutta or Butwal or Hetauda or Bhaktapur or Janakpur or Dhangadhi or Itahari or Triyuga or Siddharthanagar or Nepalganj or Madhyapur Thimi or Mechinagar or Ghorahi or Lekhnath or Kirtipur or Birendranagar or Gulariya or Tikapur or Ratnanagar or Tulsipur or kalaiya or Kamalamai or Damak or Gorkha or Rajbiraj or Kapilvastu or Byas or Lahan or Putalibazar or Panauti or Gaur or Dipayal-Silgadhi or Inaruwa or Siraha or Ramgram or Jaleswar or Baglung or Tansen or Khandbari or Bhimeshwar or Dhankuta or Bidur or Waling or Narayan or Malangwa or Bhadrapur or Amaragadhi or Dasharathchand or Ilam or Banepa or Dhulikhel or Bagmati or Gandaki or Karnali or Sudurpaschim or Patan or Birgunj or Mahendranagar).mp. |
| 39 | (Karachi or Lahore or Faisalabad or Hyderabad or Rawalpindi or Gujranwala or Peshawar or Multan or Islamabad or Quetta or Sargodha or Bahawalpur or Sialkot or Sukkur or Larkana or Sheikhupura or Rahim Yar Khan or Jhang or Dera Ghazi Khan or Gujrat or Sahiwal or Wah Cantonment or Mardan or Kasur or Okara or Mingora or Nawabshah or Chiniot or Kotri or Kamoke or Hafizabad or Sadiqabad or Mirpur Khas or Burewala or Kohat or Khanewal or Dera Ismail Khan or Turbat or Muzaffargarh or Abbotabad or Mandi Bahauddin or Shikarpur or Jacobabad or Jhelum or Khanpur or Khairpur or Khuzdar or Pakpattan or Daska or Gojra or Dadu or Muridke or Bahawalnagar or Samundri or Tando Allahyar or Tando Adam or Jaranwala or Chishtian or Attock or Vehari or Kot Abdul Malik or Ferozwala or Gwadar ir Chakwal or Gujranwala Cantonment or Kamalia or Umerkot or Ahmedpur East or Kot Addu or Wazirabad or Mansehra or Layyah or Swabi or Chaman or Taxila or Nowshera or Khushab or Shahdadkot or Mianwali or Kabal or Lodhran or Hasilpur or Charsadda or Bhakkar or Badin or Arif Wala or Ghotki or Sambrial or Jatoi or Haroonabad or Daharki or Narowal or Tando Muhammad Khan or Kamber Ali Khan or Mirpur Mathelo or Kandhkot or Bhalwal or Azad Jammu or Kashmir or Balochistan or Gilgit Baltistan or Khyber Pakhtunkhwa or Punjab or Sindh).mp. |
| 40 | (Jakarta or Surabaya or Bekasi or Bandung or Medan or Depok or Tangerang or Palembang or Semarang or Makassar or South Tangerang or Batam or Bandar Lampung or Bogor or Pekanbaru or Padang or Malang or Samarinda or Denpasar or Tasikmalaya or Serang or Balikpapan or Pontianak or Banjarmasin or Jambi or Cimahi or Surakarta or Manado or Kupang or Cilegon or Mataram or Jayapura or Bengkulu or Yogyakarta or Palu or Ambon or Sukabumi or Kendari or Cirebon or Dumai or Pekalongan or Palangka Raya or Binjai or Kediri or Sorong or Tegal or Pematangsiantar or Banjarbaru or Banda Aceh or Tarakan or Probolinggo or Singkawang or Lubuklinggau or Tanjungpinang or Bitung or Padang Sidempuan or Pangkalpinang or Batu or Pasuruan or Ternate or Banjar or Gorontalo or Madiun or Prabumulih or Salatiga or Lhokseumawe or Langsa or Palopo or Bontang or Tanjungbalai or Tebing Tinggi or Bau Bau or Bima or Parepare or Blitar or Pagar Alam or Payakumbuh or Gunungsitoli or Mojokerto or Kotamobagu or Magelang or Bukittinggi or Tidore or Tomohon or Sungai Penuh or Pariaman or Subulussalam or Sibolga or Tual or Solok or Sawahlunto or Padang Panjang or Sabang or Java or Sumatra or Sulawesi or Kalimantan or Lesser Sunda Islands or Western New Guinea or Maluku Islands).mp. |
| 41 | (Kuala Lumpur or Seberang Perai or Subang Jaya or George Town or Ipoh or Petaling Jaya or Shah Alam or Iskandar Puteri or Johor Bahru or Malacca or Kota Kinabalu or Kuantan or Alor Setar or Kuala Terengganu or Kuching or Seremban or Miri or Pasir Gudang or Penang or Selangor or Perak or Johor or Sabah or Pahang or Kedah or Terengganu or Sarawak or Negeri Sembilan).mp. |
| 42 | (Bangkok or Nonthaburi or Pak Kret or Hat Yai or Chaophraya Surasak or Surat Thani or Nakhon Ratchasima or Chiang Mai or Udon Thani or Pattaya or Khon Kaen or Nakhon Si Thammarat or Laem Chabang or Rangsit or Nakhon Sawan or Phuket or Chiang Rai or Ubon Ratchathani or Nakhon Pathom or Ko Samui or Samut Sakhon or Phitsanulok or Rayong or Songkhla or Yala or Trang or Om Noi or Sakon Nakhon or Lampang or Samut Prakan or Phra Nakhon Si Ayutthaya or Mae Sot).mp. |
| 43 | (Ho Chi Minh City or Hanoi or Haiphong or Can Thu or Da Nang or Bien Hoa or Thu Duc or "Thuan An" or Hai Duong or Dong Nai or Thua Thien Hue or Binh Duong or Nha Trang or "Di An" or Buon Ma Thuot or Thanh Hoa or Vung Tau or Thai Nguyen or Vinh or Thu Dau Mot or "Ha Long" or Quy Nhon or Khanh Hoa or Dak Lak or Ba Ria Vung Tau or "Nghe An" or Quang Ninh or Binh Dinh).mp. |
| 44 | 35 or 36 or 37 or 38 or 39 or 40 or 41 or 42 or 43 |
| 45 | 20 and 44 |
| 46 | 26 or 34 or 45 |
| 47 | 33 or 44 |
| 48 | 5 and 8 and 47 |

*Note:* This search strategy was developed for MEDLINE

| **#** | **Search history (**Embase) |
| --- | --- |
| 1 | non insulin dependent diabetes mellitus/ |
| 2 | (diabet* adj1 (type 2 or type ii)).mp. |
| 3 | (T2DM or Tiidm or niddm).mp. |
| 4 | (noninsulin depend* or non-insulin depend* or mature-onset diabet* or adult-onset diabet*).mp. |
| 5 | 1 or 2 or 3 or 4 |
| 6 | education/ or education program/ or educational model/ or educational theory/ or health education/ or "outcome of education"/ or virtual learning environment/ or health literacy/ or health promotion/ or nutrition education/ or patient education/ or consumer health information/ or distance learning/ |
| 7 | (education or learning).mp. |
| 8 | 6 or 7 |
| 9 | Care Model?.mp. |
| 10 | "model* of care".mp. |
| 11 | (healthcare model? or "model? of healthcare").mp. |
| 12 | 9 or 10 or 11 |
| 13 | 5 and 12 |
| 14 | self care/ or self help/ or self medication/ or self-testing/ or self monitoring/ or drug self administration/ or blood glucose monitoring/ |
| 15 | (self adj (care or monitor* or test* or medication* or administ* or manag* or treat* or help)).mp. |
| 16 | ((patient or self) adj (decision* or choice or determin*)).mp. |
| 17 | 14 or 15 or 16 |
| 18 | 5 and 17 |
| 19 | (model or models).mp. |
| 20 | 18 and 19 |
| 21 | 13 or 20 |
| 22 | Developing Countries/ |
| 23 | (indonesia* or malaysia* or thailand* or vietnam* or bangladesh* or india or nepal* or pakistan* or sri lanka*).mp. |
| 24 | (Mumbai or Delhi or Bangalore or Hyderabad or Ahmedabad or Chennai or Kolkata or Surat or Pune or Jaipur or Lucknow or Kanpur or Nagpur or Indore or Thane or Bhopal or Visakhapatnam or Pimpri Chinchwad or Patna or Vadodara or Ghaziabad or Ludhiana or Agra or Nashik or Faridabad or Meerut or Rajkot or Kalyan Dombivli or Vasai Virar or Varanasi or Srinagar or Aurangabad or Dhanbad or Amritsar or Navi Mumbai or Allahabad or Howrah or Ranchi or Gwalior or Jabalpur or Coimbatore or Vijayawada or Jodhpur or Madurai or Raipur or Kota or Chandigarh or Guwahati or Solapur or Hubli Dharwad or Tiruchirappalli or Bareilly or Mysore or Tiruppur or Gurgaon or Aligarh or Jalandhar or Bhubaneswar or Salem or Mira Bhayandar or Warangal or Jalgaon or Guntur or Bhiwandi or Saharanpur or Gorakhpur or Bikaner or Amravati or Noida or Jamshedpur or Bhilai or Cuttack or Firozabad or Kochi or Nellore or Bhavnagar or Dehradun or Durgapur or Asansol or Rourkela or Nanded or Kolhapur or Ajmer or Akola or Gulbarga or Jamnagar or Ujjain or Loni or Siliguri or Jhansi or Ulhasnagar or Jammu or Sangli Miraj or Kupwad or Mangalore or Erode or Belgaum or Ambattur or Tirunelveli or Malegaon or Gaya or Thiruvananthapuram or Udaipur or Maheshtala or Davanagere or Kozhikode or Kurnool or Rajpur Sonarpur or Rajahmundry or Bokaro or South Dumdum or Bellary or Patiala or Gopalpur or Agartala or Bhagalpur or Muzaffarnagar or Bhatpara or Panihati or Latur or Dhule or Tirupati or Rohtak or Korba or Bhilwara or Berhampur or Muzaffarpur or Ahmednagar or Mathura or Kollam or Avadi or Kadapa or Kamarhati or Sambalpur or Bilaspur or Shahjahanpur or Satara or Bijapur or Kakinada or Rampur or Shimoga or Chandrapur or Junagadh or Thrissur or Alwar or Bardhaman or Kulti or Nizamabad or Parbhani or Tumkur or Khammam or Ozhukarai or Bihar Sharif or Panipat or Darbhanga or Bally or Aizawl or Dewas or Ichalkaranji or Karnal or Bathinda or Jalna or Eluru or Barasat or Kirari Suleman Nagar or Purnia or Satna or Mau or Sonipat or Farrukhabad or Sagar or Durg or Imphal or Ratlam or Hapur or Arrah or Anantapur or Karimnagar or Etawah or Ambarnath or North Dumdum or Bharatpur or Begusarai or New Delhi or Gandhidham or Baranagar or Tiruvottiyur or Pondicherry or Sikar or Thoothukudi or Rewa or Mirzapur or Raichur or Pali or Ramagundam or Haridwar or Vijayanagaram or Katihar or Nagercoil or Sri Ganganagar or Karawal Nagar or Mango or Thanjavur or Bulandshahr or Uluberia or Katni or Sambhal or Singrauli or Nadiad or Secunderabad or Naihati or Yamunanagar or Bidhannagar or Pallavaram or Bidar or Munger or Panchkula or Burhanpur or Raurkela or Kharagpur or Dindigul or Gandhinagar or Hospet or Nangloi Jat or Malda or Ongole or Deoghar or Chapra or Haldia or Khandwa or Nandyal or Morena or Amroha or Anand or Bhind or Bhalswa Jahangir Pur or Madhyamgram or Bhiwani or Berhampore or Ambala or Morbi or Fatehpur or Raebareli or Mahaboobnagar or Chittoor or Bhusawal or Orai or Bahraich or Vellore or Mehsana or Raiganj or Sirsa or Danapur or Serampore or Sultan Pur Majra or Guna or Jaunpur or Panvel or Shivpuri or Surendranagar or Dudhrej or Unnao or Chinsurah or Alappuzha or Kottayam or Machilipatnam or Shimla or Adoni or Udupi or Tenali or Proddatur or Saharsa or Hindupur or Sasaram or Hajipur or Bhimavaram or Kumbakonam or Dehri or Madanapalle or Siwan or Bettiah or Guntakal or Srikakulam or Motihari or Dharmavaram or Gudivada or Phagwara or Narasaraopet or Suryapet or Miryalaguda or Tadipatri or Karaikudi or Kishanganj or Jamalpur or Ballia or Kavali or Tadepalligudem or Amaravati or Buxar or Jehanabad or Aurangabad or Gangtok).mp. |
| 25 | (Sabaragamuwa or Uva province or Kandy or Trincomalee or Anuradhapura or Jaffna or Kurunegala or Ratnapura or Galle or Badulla or Colombo or Ampara or Batticaloa or Gampaha or Hambantota or Kalutara or Kegalle or Kilinochchi or Mannar or Matale or Matara or Monaragala or Mullaitivu or Nuwara Eliya or Polonnaruwa or Puttalam or Vavuniya or Dehiwala or Moratuwa or Sri Jayawardenapura or Kotte or Negombo or Kalmunai or Vavuniya or Katunayake or Dambulla or Kolonnawa or Anuradhapura).mp. |
| 26 | (Dhaka or Chittagongor Rajshahi or Khulna or Sylhet or Mymensingh or Barisal or Rangpur or Comilla or Narayanganj or Gazipur or Bogra or Kushtia or Jessore or Cox's Bazar or Manikganj or Brahmanbaria or Dinajpur or Nawabganj or Tangail or Sirajganj or Chandpur or Feni or Jamalpur or Naogaon or Narsingdi or Pabna or Maijdee or Faridpur or Tongi or Rangpur or Savar or Siddhirganj or Jessore).mp. |
| 27 | (Kathmandu or Pokhara or Lalitpur or Biratnagar or Birganj or Dharan or Bharatpur or Bhim Dutta or Butwal or Hetauda or Bhaktapur or Janakpur or Dhangadhi or Itahari or Triyuga or Siddharthanagar or Nepalganj or Madhyapur Thimi or Mechinagar or Ghorahi or Lekhnath or Kirtipur or Birendranagar or Gulariya or Tikapur or Ratnanagar or Tulsipur or kalaiya or Kamalamai or Damak or Gorkha or Rajbiraj or Kapilvastu or Byas or Lahan or Putalibazar or Panauti or Gaur or Dipayal-Silgadhi or Inaruwa or Siraha or Ramgram or Jaleswar or Baglung or Tansen or Khandbari or Bhimeshwar or Dhankuta or Bidur or Waling or Narayan or Malangwa or Bhadrapur or Amaragadhi or Dasharathchand or Ilam or Banepa or Dhulikhel or Bagmati or Gandaki or Karnali or Sudurpaschim or Patan or Birgunj or Mahendranagar).mp. |
| 28 | (Karachi or Lahore or Faisalabad or Hyderabad or Rawalpindi or Gujranwala or Peshawar or Multan or Islamabad or Quetta or Sargodha or Bahawalpur or Sialkot or Sukkur or Larkana or Sheikhupura or Rahim Yar Khan or Jhang or Dera Ghazi Khan or Gujrat or Sahiwal or Wah Cantonment or Mardan or Kasur or Okara or Mingora or Nawabshah or Chiniot or Kotri or Kamoke or Hafizabad or Sadiqabad or Mirpur Khas or Burewala or Kohat or Khanewal or Dera Ismail Khan or Turbat or Muzaffargarh or Abbotabad or Mandi Bahauddin or Shikarpur or Jacobabad or Jhelum or Khanpur or Khairpur or Khuzdar or Pakpattan or Daska or Gojra or Dadu or Muridke or Bahawalnagar or Samundri or Tando Allahyar or Tando Adam or Jaranwala or Chishtian or Attock or Vehari or Kot Abdul Malik or Ferozwala or Gwadar ir Chakwal or Gujranwala Cantonment or Kamalia or Umerkot or Ahmedpur East or Kot Addu or Wazirabad or Mansehra or Layyah or Swabi or Chaman or Taxila or Nowshera or Khushab or Shahdadkot or Mianwali or Kabal or Lodhran or Hasilpur or Charsadda or Bhakkar or Badin or Arif Wala or Ghotki or Sambrial or Jatoi or Haroonabad or Daharki or Narowal or Tando Muhammad Khan or Kamber Ali Khan or Mirpur Mathelo or Kandhkot or Bhalwal or Azad Jammu or Kashmir or Balochistan or Gilgit Baltistan or Khyber Pakhtunkhwa or Punjab or Sindh).mp. |
| 29 | (Jakarta or Surabaya or Bekasi or Bandung or Medan or Depok or Tangerang or Palembang or Semarang or Makassar or South Tangerang or Batam or Bandar Lampung or Bogor or Pekanbaru or Padang or Malang or Samarinda or Denpasar or Tasikmalaya or Serang or Balikpapan or Pontianak or Banjarmasin or Jambi or Cimahi or Surakarta or Manado or Kupang or Cilegon or Mataram or Jayapura or Bengkulu or Yogyakarta or Palu or Ambon or Sukabumi or Kendari or Cirebon or Dumai or Pekalongan or Palangka Raya or Binjai or Kediri or Sorong or Tegal or Pematangsiantar or Banjarbaru or Banda Aceh or Tarakan or Probolinggo or Singkawang or Lubuklinggau or Tanjungpinang or Bitung or Padang Sidempuan or Pangkalpinang or Batu or Pasuruan or Ternate or Banjar or Gorontalo or Madiun or Prabumulih or Salatiga or Lhokseumawe or Langsa or Palopo or Bontang or Tanjungbalai or Tebing Tinggi or Bau Bau or Bima or Parepare or Blitar or Pagar Alam or Payakumbuh or Gunungsitoli or Mojokerto or Kotamobagu or Magelang or Bukittinggi or Tidore or Tomohon or Sungai Penuh or Pariaman or Subulussalam or Sibolga or Tual or Solok or Sawahlunto or Padang Panjang or Sabang or Java or Sumatra or Sulawesi or Kalimantan or Lesser Sunda Islands or Western New Guinea or Maluku Islands).mp. |
| 30 | (Kuala Lumpur or Seberang Perai or Subang Jaya or George Town or Ipoh or Petaling Jaya or Shah Alam or Iskandar Puteri or Johor Bahru or Malacca or Kota Kinabalu or Kuantan or Alor Setar or Kuala Terengganu or Kuching or Seremban or Miri or Pasir Gudang or Penang or Selangor or Perak or Johor or Sabah or Pahang or Kedah or Terengganu or Sarawak or Negeri Sembilan).mp. |
| 31 | (Bangkok or Nonthaburi or Pak Kret or Hat Yai or Chaophraya Surasak or Surat Thani or Nakhon Ratchasima or Chiang Mai or Udon Thani or Pattaya or Khon Kaen or Nakhon Si Thammarat or Laem Chabang or Rangsit or Nakhon Sawan or Phuket or Chiang Rai or Ubon Ratchathani or Nakhon Pathom or Ko Samui or Samut Sakhon or Phitsanulok or Rayong or Songkhla or Yala or Trang or Om Noi or Sakon Nakhon or Lampang or Samut Prakan or Phra Nakhon Si Ayutthaya or Mae Sot).mp. |
| 32 | (Ho Chi Minh City or Hanoi or Haiphong or Can Thu or Da Nang or Bien Hoa or Thu Duc or "Thuan An" or Hai Duong or Dong Nai or Thua Thien Hue or Binh Duong or Nha Trang or "Di An" or Buon Ma Thuot or Thanh Hoa or Vung Tau or Thai Nguyen or Vinh or Thu Dau Mot or "Ha Long" or Quy Nhon or Khanh Hoa or Dak Lak or Ba Ria Vung Tau or "Nghe An" or Quang Ninh or Binh Dinh).mp. |
| 33 | ((developing or low* income or middle income) adj (countr* or nation?)).mp. |
| 34 | ((developing or third) adj world).mp. |
| 35 | (resource-limit* or resource-poor or low-resource* or limited-resource* or resource-constrain* or constrain*-resource* or under-resource* or poor*-resource* or resource-scarce* or scarce*-resource* or low-income or middle-income or lowincome or middleincome or (low adj3 middle-income)).mp. |
| 36 | ((developing or underdeveloped or under-developed or emerging or less-developed or least-developed or less-economically developed or least-economically developed or less-affluent or least-affluent or deprived or poor) adj (country or countries or nation or nations or region or regions or economy or economies)).mp. |
| 37 | ((developing or underdeveloped or under-developed or less-developed or least-developed) adj (population* or world)).mp. |
| 38 | (third-world* or thirdworld* or 3rd-world* or lmic or lmics or lami countr* or lalmi countr* or transitional countr*).mp. |
| 39 | (low* adj (gdp or gnp or gross domestic or gross national)).mp. |
| 40 | ((underserved or under served) adj (countr* or nation? or population*)).mp. |
| 41 | 22 or 23 or 24 or 25 or 26 or 27 or 28 or 29 or 30 or 31 or 32 or 33 or 34 or 35 or 36 or 37 or 38 or 39 or 40 |
| 42 | 21 and 41 |
| 43 | 5 and 8 and 41 |
| 44 | diabetes education/ |
| 45 | 41 and 44 |
| 46 | 43 or 45 |

*Note:* This search strategy was developed for Embase

| **#** | **Search history (Cinahl)** |
| --- | --- |
| S45 | S42 OR S44 |
| S44 | S41 AND S43 |
| S43 | (MH "Diabetes Education") |
| S42 | S5 AND S40 AND S41 |
| S41 | S26 OR S37 |
| S40 | ( (MH "Health Information") OR (MH "Consumer Health Information") OR (MH "Health Literacy") ) OR ( (MH "Education") OR (MH "Online Education") OR (MH "Nutrition Education") OR (MH "Patient Education") OR (MH "Health Education") OR (MH "Adult Education") ) OR ( (education or learning) ) |
| S39 | S27 OR S38 |
| S38 | S17 AND S37 |
| S37 | S28 OR S29 OR S30 OR S31 OR S32 OR S33 OR S34 OR S35 OR S36 |
| S36 | (Ho Chi Minh City or Hanoi or Haiphong or Can Thu or Da Nang or Bien Hoa or Thu Duc or "Thuan An" or Hai Duong or Dong Nai or Thua Thien Hue or Binh Duong or Nha Trang or "Di An" or Buon Ma Thuot or Thanh Hoa or Vung Tau or Thai Nguyen or Vinh or Thu Dau Mot or "Ha Long" or Quy Nhon or Khanh Hoa or Dak Lak or Ba Ria Vung Tau or "Nghe An" or Quang Ninh or Binh Dinh) |
| S35 | (Bangkok or Nonthaburi or Pak Kret or Hat Yai or Chaophraya Surasak or Surat Thani or Nakhon Ratchasima or Chiang Mai or Udon Thani or Pattaya or Khon Kaen or Nakhon Si Thammarat or Laem Chabang or Rangsit or Nakhon Sawan or Phuket or Chiang Rai or Ubon Ratchathani or Nakhon Pathom or Ko Samui or Samut Sakhon or Phitsanulok or Rayong or Songkhla or Yala or Trang or Om Noi or Sakon Nakhon or Lampang or Samut Prakan or Phra Nakhon Si Ayutthaya or Mae Sot) |
| S34 | (Kuala Lumpur or Seberang Perai or Subang Jaya or George Town or Ipoh or Petaling Jaya or Shah Alam or Iskandar Puteri or Johor Bahru or Malacca or Kota Kinabalu or Kuantan or Alor Setar or Kuala Terengganu or Kuching or Seremban or Miri or Pasir Gudang or Penang or Selangor or Perak or Johor or Sabah or Pahang or Kedah or Terengganu or Sarawak or Negeri Sembilan) |
| S33 | (Jakarta or Surabaya or Bekasi or Bandung or Medan or Depok or Tangerang or Palembang or Semarang or Makassar or South Tangerang or Batam or Bandar Lampung or Bogor or Pekanbaru or Padang or Malang or Samarinda or Denpasar or Tasikmalaya or Serang or Balikpapan or Pontianak or Banjarmasin or Jambi or Cimahi or Surakarta or Manado or Kupang or Cilegon or Mataram or Jayapura or Bengkulu or Yogyakarta or Palu or Ambon or Sukabumi or Kendari or Cirebon or Dumai or Pekalongan or Palangka Raya or Binjai or Kediri or Sorong or Tegal or Pematangsiantar or Banjarbaru or Banda Aceh or Tarakan or Probolinggo or Singkawang or Lubuklinggau or Tanjungpinang or Bitung or Padang Sidempuan or Pangkalpinang or Batu or Pasuruan or Ternate or Banjar or Gorontalo or Madiun or Prabumulih or Salatiga or Lhokseumawe or Langsa or Palopo or Bontang or Tanjungbalai or Tebing Tinggi or Bau Bau or Bima or Parepare or Blitar or Pagar Alam or Payakumbuh or Gunungsitoli or Mojokerto or Kotamobagu or Magelang or Bukittinggi or Tidore or Tomohon or Sungai Penuh or Pariaman or Subulussalam or Sibolga or Tual or Solok or Sawahlunto or Padang Panjang or Sabang or Java or Sumatra or Sulawesi or Kalimantan or Lesser Sunda Islands or Western New Guinea or Maluku Islands) |
| S32 | (Karachi or Lahore or Faisalabad or Hyderabad or Rawalpindi or Gujranwala or Peshawar or Multan or Islamabad or Quetta or Sargodha or Bahawalpur or Sialkot or Sukkur or Larkana or Sheikhupura or Rahim Yar Khan or Jhang or Dera Ghazi Khan or Gujrat or Sahiwal or Wah Cantonment or Mardan or Kasur or Okara or Mingora or Nawabshah or Chiniot or Kotri or Kamoke or Hafizabad or Sadiqabad or Mirpur Khas or Burewala or Kohat or Khanewal or Dera Ismail Khan or Turbat or Muzaffargarh or Abbotabad or Mandi Bahauddin or Shikarpur or Jacobabad or Jhelum or Khanpur or Khairpur or Khuzdar or Pakpattan or Daska or Gojra or Dadu or Muridke or Bahawalnagar or Samundri or Tando Allahyar or Tando Adam or Jaranwala or Chishtian or Attock or Vehari or Kot Abdul Malik or Ferozwala or Gwadar ir Chakwal or Gujranwala Cantonment or Kamalia or Umerkot or Ahmedpur East or Kot Addu or Wazirabad or Mansehra or Layyah or Swabi or Chaman or Taxila or Nowshera or Khushab or Shahdadkot or Mianwali or Kabal or Lodhran or Hasilpur or Charsadda or Bhakkar or Badin or Arif Wala or Ghotki or Sambrial or Jatoi or Haroonabad or Daharki or Narowal or Tando Muhammad Khan or Kamber Ali Khan or Mirpur Mathelo or Kandhkot or Bhalwal or Azad Jammu or Kashmir or Balochistan or Gilgit Baltistan or Khyber Pakhtunkhwa or Punjab or Sindh) |
| S31 | (Kathmandu or Pokhara or Lalitpur or Biratnagar or Birganj or Dharan or Bharatpur or Bhim Dutta or Butwal or Hetauda or Bhaktapur or Janakpur or Dhangadhi or Itahari or Triyuga or Siddharthanagar or Nepalganj or Madhyapur Thimi or Mechinagar or Ghorahi or Lekhnath or Kirtipur or Birendranagar or Gulariya or Tikapur or Ratnanagar or Tulsipur or kalaiya or Kamalamai or Damak or Gorkha or Rajbiraj or Kapilvastu or Byas or Lahan or Putalibazar or Panauti or Gaur or Dipayal-Silgadhi or Inaruwa or Siraha or Ramgram or Jaleswar or Baglung or Tansen or Khandbari or Bhimeshwar or Dhankuta or Bidur or Waling or Narayan or Malangwa or Bhadrapur or Amaragadhi or Dasharathchand or Ilam or Banepa or Dhulikhel or Bagmati or Gandaki or Karnali or Sudurpaschim or Patan or Birgunj or Mahendranagar) |
| S30 | (Dhaka or Chittagongor Rajshahi or Khulna or Sylhet or Mymensingh or Barisal or Rangpur or Comilla or Narayanganj or Gazipur or Bogra or Kushtia or Jessore or Cox's Bazar or Manikganj or Brahmanbaria or Dinajpur or Nawabganj or Tangail or Sirajganj or Chandpur or Feni or Jamalpur or Naogaon or Narsingdi or Pabna or Maijdee or Faridpur or Tongi or Rangpur or Savar or Siddhirganj or Jessore) |
| S29 | (Sabaragamuwa or Uva province or Kandy or Trincomalee or Anuradhapura or Jaffna or Kurunegala or Ratnapura or Galle or Badulla or Colombo or Ampara or Batticaloa or Gampaha or Hambantota or Kalutara or Kegalle or Kilinochchi or Mannar or Matale or Matara or Monaragala or Mullaitivu or Nuwara Eliya or Polonnaruwa or Puttalam or Vavuniya or Dehiwala or Moratuwa or Sri Jayawardenapura or Kotte or Negombo or Kalmunai or Vavuniya or Katunayake or Dambulla or Kolonnawa or Anuradhapura) |
| S28 | (Mumbai or Delhi or Bangalore or Hyderabad or Ahmedabad or Chennai or Kolkata or Surat or Pune or Jaipur or Lucknow or Kanpur or Nagpur or Indore or Thane or Bhopal or Visakhapatnam or Pimpri Chinchwad or Patna or Vadodara or Ghaziabad or Ludhiana or Agra or Nashik or Faridabad or Meerut or Rajkot or Kalyan Dombivli or Vasai Virar or Varanasi or Srinagar or Aurangabad or Dhanbad or Amritsar or Navi Mumbai or Allahabad or Howrah or Ranchi or Gwalior or Jabalpur or Coimbatore or Vijayawada or Jodhpur or Madurai or Raipur or Kota or Chandigarh or Guwahati or Solapur or Hubli Dharwad or Tiruchirappalli or Bareilly or Mysore or Tiruppur or Gurgaon or Aligarh or Jalandhar or Bhubaneswar or Salem or Mira Bhayandar or Warangal or Jalgaon or Guntur or Bhiwandi or Saharanpur or Gorakhpur or Bikaner or Amravati or Noida or Jamshedpur or Bhilai or Cuttack or Firozabad or Kochi or Nellore or Bhavnagar or Dehradun or Durgapur or Asansol or Rourkela or Nanded or Kolhapur or Ajmer or Akola or Gulbarga or Jamnagar or Ujjain or Loni or Siliguri or Jhansi or Ulhasnagar or Jammu or Sangli Miraj or Kupwad or Mangalore or Erode or Belgaum or Ambattur or Tirunelveli or Malegaon or Gaya or Thiruvananthapuram or Udaipur or Maheshtala or Davanagere or Kozhikode or Kurnool or Rajpur Sonarpur or Rajahmundry or Bokaro or South Dumdum or Bellary or Patiala or Gopalpur or Agartala or Bhagalpur or Muzaffarnagar or Bhatpara or Panihati or Latur or Dhule or Tirupati or Rohtak or Korba or Bhilwara or Berhampur or Muzaffarpur or Ahmednagar or Mathura or Kollam or Avadi or Kadapa or Kamarhati or Sambalpur or Bilaspur or Shahjahanpur or Satara or Bijapur or Kakinada or Rampur or Shimoga or Chandrapur or Junagadh or Thrissur or Alwar or Bardhaman or Kulti or Nizamabad or Parbhani or Tumkur or Khammam or Ozhukarai or Bihar Sharif or Panipat or Darbhanga or Bally or Aizawl or Dewas or Ichalkaranji or Karnal or Bathinda or Jalna or Eluru or Barasat or Kirari Suleman Nagar or Purnia or Satna or Mau or Sonipat or Farrukhabad or Sagar or Durg or Imphal or Ratlam or Hapur or Arrah or Anantapur or Karimnagar or Etawah or Ambarnath or North Dumdum or Bharatpur or Begusarai or New Delhi or Gandhidham or Baranagar or Tiruvottiyur or Pondicherry or Sikar or Thoothukudi or Rewa or Mirzapur or Raichur or Pali or Ramagundam or Haridwar or Vijayanagaram or Katihar or Nagercoil or Sri Ganganagar or Karawal Nagar or Mango or Thanjavur or Bulandshahr or Uluberia or Katni or Sambhal or Singrauli or Nadiad or Secunderabad or Naihati or Yamunanagar or Bidhannagar or Pallavaram or Bidar or Munger or Panchkula or Burhanpur or Raurkela or Kharagpur or Dindigul or Gandhinagar or Hospet or Nangloi Jat or Malda or Ongole or Deoghar or Chapra or Haldia or Khandwa or Nandyal or Morena or Amroha or Anand or Bhind or Bhalswa Jahangir Pur or Madhyamgram or Bhiwani or Berhampore or Ambala or Morbi or Fatehpur or Raebareli or Mahaboobnagar or Chittoor or Bhusawal or Orai or Bahraich or Vellore or Mehsana or Raiganj or Sirsa or Danapur or Serampore or Sultan Pur Majra or Guna or Jaunpur or Panvel or Shivpuri or Surendranagar or Dudhrej or Unnao or Chinsurah or Alappuzha or Kottayam or Machilipatnam or Shimla or Adoni or Udupi or Tenali or Proddatur or Saharsa or Hindupur or Sasaram or Hajipur or Bhimavaram or Kumbakonam or Dehri or Madanapalle or Siwan or Bettiah or Guntakal or Srikakulam or Motihari or Dharmavaram or Gudivada or Phagwara or Narasaraopet or Suryapet or Miryalaguda or Tadipatri or Karaikudi or Kishanganj or Jamalpur or Ballia or Kavali or Tadepalligudem or Amaravati or Buxar or Jehanabad or Aurangabad or Gangtok) |
| S27 | S17 AND S26 |
| S26 | S18 OR S19 OR S20 OR S21 OR S22 OR S23 OR S24 OR S25 |
| S25 | ((underserved or under served) N0 (countr* or nation? or population*)) |
| S24 | (low* N0 (gdp or gnp or gross domestic or gross national)) |
| S23 | (third-world* or thirdworld* or 3rd-world* or lmic or lmics or lami countr* or lalmi countr* or transitional countr*) |
| S22 | ((developing or underdeveloped or under-developed or less-developed or least-developed) N0 (population* or world)) |
| S21 | ((developing or underdeveloped or under-developed or emerging or less-developed or least-developed or less-economically developed or least-economically developed or less-affluent or least-affluent or deprived or poor) N0 (country or countries or nation or nations or region or regions or economy or economies)) |
| S20 | ("resource-limit*" or "resource-poor" or "low-resource*" or "limited-resource*" or "resource-constrain*" or "constrain*-resource*" or "under-resource*" or "poor*-resource*" or "resource-scarce*" or "scarce*-resource*" or "low-income" or "middle-income" or "lowincome" or "middleincome" or (low N3 "middle-income")) |
| S19 | ( (MH "India") OR (MH "Bhutan") OR (MH "Pakistan") OR (MH "Nepal") OR (MH "Sri Lanka") ) OR ( (MH "Vietnam") OR (MH "Indonesia") OR (MH "Malaysia") OR (MH "Thailand") ) |
| S18 | (MH "Developing Countries") |
| S17 | S9 OR S16 |
| S16 | S14 AND S15 |
| S15 | (model or models) |
| S14 | S5 AND S13 |
| S13 | S10 OR S11 OR S12 |
| S12 | ((patient or self) N0 (decision* or choice or determin*)) |
| S11 | (self N0 (care or monitor* or test* or medication* or administ* or manag*)) |
| S10 | ((MH "Self Care") OR (MH "Blood Glucose Self-Monitoring") OR (MH "Self Medication") OR (MH "Self-Management") OR (MH "Self-Testing") ) OR (MH "Self Administration") |
| S9 | S5 AND S8 |
| S8 | S6 OR S7 |
| S7 | ("model* of care" or "model of healthcare") |
| S6 | ((healthcare or care) N0 model?) |
| S5 | S1 OR S2 OR S3 OR S4 |
| S4 | (“noninsulin depend*” or “non-insulin depend*” or “mature-onset diabet*” or “adult-onset diabet*”) |
| S3 | (T2DM or Tiidm or niddm) |
| S2 | (diabet* N1 (type 2 or type ii)) |
| S1 | (MH "Diabetes Mellitus, Type 2") |

*Note:* This search strategy was developed for Cinahl

| **#** | **Search history (Global health)** |
| --- | --- |
| 1 | Diabetes Mellitus, Type 2/ |
| 2 | (diabet* adj1 (type 2 or type ii)).mp. |
| 3 | (T2DM or Tiidm or niddm).mp. |
| 4 | (noninsulin depend* or non-insulin depend* or mature-onset diabet* or adult-onset diabet*).mp. |
| 5 | 1 or 2 or 3 or 4 |
| 6 | ((healthcare or care) adj model?).mp. |
| 7 | ("model* of care" or "model of healthcare").mp. |
| 8 | 6 or 7 |
| 9 | 5 and 8 |
| 10 | self care/ or self administration/ or self medication/ or self-testing/ or blood glucose self-monitoring/ |
| 11 | (self adj (care or monitor* or test* or medication* or administ* or manag* or treat* or help)).mp. |
| 12 | ((patient or self) adj (decision* or choice or determin*)).mp. |
| 13 | 10 or 11 or 12 |
| 14 | 5 and 13 |
| 15 | (model or models).mp. |
| 16 | 14 and 15 |
| 17 | 9 or 16 |
| 18 | Developing Countries/ |
| 19 | (indonesia* or malaysia* or thailand* or vietnam* or bangladesh* or india or nepal* or pakistan* or sri lanka*).mp. |
| 20 | ((developing or low* income or middle income) adj (countr* or nation?)).mp. |
| 21 | ((developing or third) adj world).mp. |
| 22 | 18 or 19 or 20 or 21 |
| 23 | 17 and 22 |
| 24 | (resource-limit* or resource-poor or low-resource* or limited-resource* or resource-constrain* or constrain*-resource* or under-resource* or poor*-resource* or resource-scarce* or scarce*-resource* or low-income or middle-income or lowincome or middleincome or (low adj3 middle-income)).mp. |
| 25 | ((developing or underdeveloped or under-developed or emerging or less-developed or least-developed or less-economically developed or least-economically developed or less-affluent or least-affluent or deprived or poor) adj (country or countries or nation or nations or region or regions or economy or economies)).mp. |
| 26 | ((developing or underdeveloped or under-developed or less-developed or least-developed) adj (population* or world)).mp. |
| 27 | (third-world* or thirdworld* or 3rd-world* or lmic or lmics or lami countr* or lalmi countr* or transitional countr*).mp. |
| 28 | (low* adj (gdp or gnp or gross domestic or gross national)).mp. |
| 29 | ((underserved or under served) adj (countr* or nation? or population*)).mp. |
| 30 | 22 or 24 or 25 or 26 or 27 or 28 or 29 |
| 31 | 17 and 30 |
| 32 | (Mumbai or Delhi or Bangalore or Hyderabad or Ahmedabad or Chennai or Kolkata or Surat or Pune or Jaipur or Lucknow or Kanpur or Nagpur or Indore or Thane or Bhopal or Visakhapatnam or Pimpri Chinchwad or Patna or Vadodara or Ghaziabad or Ludhiana or Agra or Nashik or Faridabad or Meerut or Rajkot or Kalyan Dombivli or Vasai Virar or Varanasi or Srinagar or Aurangabad or Dhanbad or Amritsar or Navi Mumbai or Allahabad or Howrah or Ranchi or Gwalior or Jabalpur or Coimbatore or Vijayawada or Jodhpur or Madurai or Raipur or Kota or Chandigarh or Guwahati or Solapur or Hubli Dharwad or Tiruchirappalli or Bareilly or Mysore or Tiruppur or Gurgaon or Aligarh or Jalandhar or Bhubaneswar or Salem or Mira Bhayandar or Warangal or Jalgaon or Guntur or Bhiwandi or Saharanpur or Gorakhpur or Bikaner or Amravati or Noida or Jamshedpur or Bhilai or Cuttack or Firozabad or Kochi or Nellore or Bhavnagar or Dehradun or Durgapur or Asansol or Rourkela or Nanded or Kolhapur or Ajmer or Akola or Gulbarga or Jamnagar or Ujjain or Loni or Siliguri or Jhansi or Ulhasnagar or Jammu or Sangli Miraj or Kupwad or Mangalore or Erode or Belgaum or Ambattur or Tirunelveli or Malegaon or Gaya or Thiruvananthapuram or Udaipur or Maheshtala or Davanagere or Kozhikode or Kurnool or Rajpur Sonarpur or Rajahmundry or Bokaro or South Dumdum or Bellary or Patiala or Gopalpur or Agartala or Bhagalpur or Muzaffarnagar or Bhatpara or Panihati or Latur or Dhule or Tirupati or Rohtak or Korba or Bhilwara or Berhampur or Muzaffarpur or Ahmednagar or Mathura or Kollam or Avadi or Kadapa or Kamarhati or Sambalpur or Bilaspur or Shahjahanpur or Satara or Bijapur or Kakinada or Rampur or Shimoga or Chandrapur or Junagadh or Thrissur or Alwar or Bardhaman or Kulti or Nizamabad or Parbhani or Tumkur or Khammam or Ozhukarai or Bihar Sharif or Panipat or Darbhanga or Bally or Aizawl or Dewas or Ichalkaranji or Karnal or Bathinda or Jalna or Eluru or Barasat or Kirari Suleman Nagar or Purnia or Satna or Mau or Sonipat or Farrukhabad or Sagar or Durg or Imphal or Ratlam or Hapur or Arrah or Anantapur or Karimnagar or Etawah or Ambarnath or North Dumdum or Bharatpur or Begusarai or New Delhi or Gandhidham or Baranagar or Tiruvottiyur or Pondicherry or Sikar or Thoothukudi or Rewa or Mirzapur or Raichur or Pali or Ramagundam or Haridwar or Vijayanagaram or Katihar or Nagercoil or Sri Ganganagar or Karawal Nagar or Mango or Thanjavur or Bulandshahr or Uluberia or Katni or Sambhal or Singrauli or Nadiad or Secunderabad or Naihati or Yamunanagar or Bidhannagar or Pallavaram or Bidar or Munger or Panchkula or Burhanpur or Raurkela or Kharagpur or Dindigul or Gandhinagar or Hospet or Nangloi Jat or Malda or Ongole or Deoghar or Chapra or Haldia or Khandwa or Nandyal or Morena or Amroha or Anand or Bhind or Bhalswa Jahangir Pur or Madhyamgram or Bhiwani or Berhampore or Ambala or Morbi or Fatehpur or Raebareli or Mahaboobnagar or Chittoor or Bhusawal or Orai or Bahraich or Vellore or Mehsana or Raiganj or Sirsa or Danapur or Serampore or Sultan Pur Majra or Guna or Jaunpur or Panvel or Shivpuri or Surendranagar or Dudhrej or Unnao or Chinsurah or Alappuzha or Kottayam or Machilipatnam or Shimla or Adoni or Udupi or Tenali or Proddatur or Saharsa or Hindupur or Sasaram or Hajipur or Bhimavaram or Kumbakonam or Dehri or Madanapalle or Siwan or Bettiah or Guntakal or Srikakulam or Motihari or Dharmavaram or Gudivada or Phagwara or Narasaraopet or Suryapet or Miryalaguda or Tadipatri or Karaikudi or Kishanganj or Jamalpur or Ballia or Kavali or Tadepalligudem or Amaravati or Buxar or Jehanabad or Aurangabad or Gangtok).mp. |
| 33 | (Sabaragamuwa or Uva province or Kandy or Trincomalee or Anuradhapura or Jaffna or Kurunegala or Ratnapura or Galle or Badulla or Colombo or Ampara or Batticaloa or Gampaha or Hambantota or Kalutara or Kegalle or Kilinochchi or Mannar or Matale or Matara or Monaragala or Mullaitivu or Nuwara Eliya or Polonnaruwa or Puttalam or Vavuniya or Dehiwala or Moratuwa or Sri Jayawardenapura or Kotte or Negombo or Kalmunai or Vavuniya or Katunayake or Dambulla or Kolonnawa or Anuradhapura).mp. |
| 34 | (Dhaka or Chittagongor Rajshahi or Khulna or Sylhet or Mymensingh or Barisal or Rangpur or Comilla or Narayanganj or Gazipur or Bogra or Kushtia or Jessore or Cox's Bazar or Manikganj or Brahmanbaria or Dinajpur or Nawabganj or Tangail or Sirajganj or Chandpur or Feni or Jamalpur or Naogaon or Narsingdi or Pabna or Maijdee or Faridpur or Tongi or Rangpur or Savar or Siddhirganj or Jessore).mp. |
| 35 | (Kathmandu or Pokhara or Lalitpur or Biratnagar or Birganj or Dharan or Bharatpur or Bhim Dutta or Butwal or Hetauda or Bhaktapur or Janakpur or Dhangadhi or Itahari or Triyuga or Siddharthanagar or Nepalganj or Madhyapur Thimi or Mechinagar or Ghorahi or Lekhnath or Kirtipur or Birendranagar or Gulariya or Tikapur or Ratnanagar or Tulsipur or kalaiya or Kamalamai or Damak or Gorkha or Rajbiraj or Kapilvastu or Byas or Lahan or Putalibazar or Panauti or Gaur or Dipayal-Silgadhi or Inaruwa or Siraha or Ramgram or Jaleswar or Baglung or Tansen or Khandbari or Bhimeshwar or Dhankuta or Bidur or Waling or Narayan or Malangwa or Bhadrapur or Amaragadhi or Dasharathchand or Ilam or Banepa or Dhulikhel or Bagmati or Gandaki or Karnali or Sudurpaschim or Patan or Birgunj or Mahendranagar).mp. |
| 36 | (Karachi or Lahore or Faisalabad or Hyderabad or Rawalpindi or Gujranwala or Peshawar or Multan or Islamabad or Quetta or Sargodha or Bahawalpur or Sialkot or Sukkur or Larkana or Sheikhupura or Rahim Yar Khan or Jhang or Dera Ghazi Khan or Gujrat or Sahiwal or Wah Cantonment or Mardan or Kasur or Okara or Mingora or Nawabshah or Chiniot or Kotri or Kamoke or Hafizabad or Sadiqabad or Mirpur Khas or Burewala or Kohat or Khanewal or Dera Ismail Khan or Turbat or Muzaffargarh or Abbotabad or Mandi Bahauddin or Shikarpur or Jacobabad or Jhelum or Khanpur or Khairpur or Khuzdar or Pakpattan or Daska or Gojra or Dadu or Muridke or Bahawalnagar or Samundri or Tando Allahyar or Tando Adam or Jaranwala or Chishtian or Attock or Vehari or Kot Abdul Malik or Ferozwala or Gwadar ir Chakwal or Gujranwala Cantonment or Kamalia or Umerkot or Ahmedpur East or Kot Addu or Wazirabad or Mansehra or Layyah or Swabi or Chaman or Taxila or Nowshera or Khushab or Shahdadkot or Mianwali or Kabal or Lodhran or Hasilpur or Charsadda or Bhakkar or Badin or Arif Wala or Ghotki or Sambrial or Jatoi or Haroonabad or Daharki or Narowal or Tando Muhammad Khan or Kamber Ali Khan or Mirpur Mathelo or Kandhkot or Bhalwal or Azad Jammu or Kashmir or Balochistan or Gilgit Baltistan or Khyber Pakhtunkhwa or Punjab or Sindh).mp. |
| 37 | (Jakarta or Surabaya or Bekasi or Bandung or Medan or Depok or Tangerang or Palembang or Semarang or Makassar or South Tangerang or Batam or Bandar Lampung or Bogor or Pekanbaru or Padang or Malang or Samarinda or Denpasar or Tasikmalaya or Serang or Balikpapan or Pontianak or Banjarmasin or Jambi or Cimahi or Surakarta or Manado or Kupang or Cilegon or Mataram or Jayapura or Bengkulu or Yogyakarta or Palu or Ambon or Sukabumi or Kendari or Cirebon or Dumai or Pekalongan or Palangka Raya or Binjai or Kediri or Sorong or Tegal or Pematangsiantar or Banjarbaru or Banda Aceh or Tarakan or Probolinggo or Singkawang or Lubuklinggau or Tanjungpinang or Bitung or Padang Sidempuan or Pangkalpinang or Batu or Pasuruan or Ternate or Banjar or Gorontalo or Madiun or Prabumulih or Salatiga or Lhokseumawe or Langsa or Palopo or Bontang or Tanjungbalai or Tebing Tinggi or Bau Bau or Bima or Parepare or Blitar or Pagar Alam or Payakumbuh or Gunungsitoli or Mojokerto or Kotamobagu or Magelang or Bukittinggi or Tidore or Tomohon or Sungai Penuh or Pariaman or Subulussalam or Sibolga or Tual or Solok or Sawahlunto or Padang Panjang or Sabang or Java or Sumatra or Sulawesi or Kalimantan or Lesser Sunda Islands or Western New Guinea or Maluku Islands).mp. |
| 38 | (Kuala Lumpur or Seberang Perai or Subang Jaya or George Town or Ipoh or Petaling Jaya or Shah Alam or Iskandar Puteri or Johor Bahru or Malacca or Kota Kinabalu or Kuantan or Alor Setar or Kuala Terengganu or Kuching or Seremban or Miri or Pasir Gudang or Penang or Selangor or Perak or Johor or Sabah or Pahang or Kedah or Terengganu or Sarawak or Negeri Sembilan).mp. |
| 39 | (Bangkok or Nonthaburi or Pak Kret or Hat Yai or Chaophraya Surasak or Surat Thani or Nakhon Ratchasima or Chiang Mai or Udon Thani or Pattaya or Khon Kaen or Nakhon Si Thammarat or Laem Chabang or Rangsit or Nakhon Sawan or Phuket or Chiang Rai or Ubon Ratchathani or Nakhon Pathom or Ko Samui or Samut Sakhon or Phitsanulok or Rayong or Songkhla or Yala or Trang or Om Noi or Sakon Nakhon or Lampang or Samut Prakan or Phra Nakhon Si Ayutthaya or Mae Sot).mp. |
| 40 | (Ho Chi Minh City or Hanoi or Haiphong or Can Thu or Da Nang or Bien Hoa or Thu Duc or "Thuan An" or Hai Duong or Dong Nai or Thua Thien Hue or Binh Duong or Nha Trang or "Di An" or Buon Ma Thuot or Thanh Hoa or Vung Tau or Thai Nguyen or Vinh or Thu Dau Mot or "Ha Long" or Quy Nhon or Khanh Hoa or Dak Lak or Ba Ria Vung Tau or "Nghe An" or Quang Ninh or Binh Dinh).mp. |
| 41 | 32 or 33 or 34 or 35 or 36 or 37 or 38 or 39 or 40 |
| 42 | 17 and 41 |
| 43 | 23 or 31 or 42 |
| 44 | education/ or adult education/ or community education/ or consumer education/ or dietetic education/ or health education/ or nutrition education/ or patient education/ |
| 45 | health promotion.sh. |
| 46 | (education or learning).mp. |
| 47 | 44 or 45 or 46 |
| 48 | 22 or 30 or 41 |
| 49 | 5 and 47 and 48 |

*Note:* This search strategy was developed for global health

| **#** | **Search History (Cocrance)** |
| --- | --- |
| 1 | Diabetes Mellitus, Type 2/ |
| 2 | (diabet* adj1 (type 2 or type ii)).mp. |
| 3 | (T2DM or Tiidm or niddm).mp. |
| 4 | (noninsulin depend* or non-insulin depend* or mature-onset diabet* or adult-onset diabet*).mp. |
| 5 | 1 or 2 or 3 or 4 |
| 6 | education/ or education, distance/ or health education/ or consumer health information/ or health literacy/ or health promotion/ or patient education as topic/ |
| 7 | (education or learning).mp. |
| 8 | 6 or 7 |
| 9 | ((healthcare or care) adj model?).mp. |
| 10 | ("model* of care" or "model of healthcare").mp. |
| 11 | 9 or 10 |
| 12 | 5 and 11 |
| 13 | self care/ or self administration/ or self medication/ or self-testing/ or blood glucose self-monitoring/ |
| 14 | (self adj (care or monitor* or test* or medication* or administ* or manag* or treat* or help)).mp. |
| 15 | ((patient or self) adj (decision* or choice or determin*)).mp. |
| 16 | 13 or 14 or 15 |
| 17 | 5 and 16 |
| 18 | (model or models).mp. |
| 19 | 17 and 18 |
| 20 | 12 or 19 |
| 21 | Developing Countries/ |
| 22 | (indonesia* or malaysia* or thailand* or vietnam* or bangladesh* or india or nepal* or pakistan* or sri lanka*).mp. |
| 23 | ((developing or low* income or middle income) adj (countr* or nation?)).mp. |
| 24 | ((developing or third) adj world).mp. |
| 25 | 21 or 22 or 23 or 24 |
| 26 | 20 and 25 |
| 27 | (resource-limit* or resource-poor or low-resource* or limited-resource* or resource-constrain* or constrain*-resource* or under-resource* or poor*-resource* or resource-scarce* or scarce*-resource* or low-income or middle-income or lowincome or middleincome or (low adj3 middle-income)).mp. |
| 28 | ((developing or underdeveloped or under-developed or emerging or less-developed or least-developed or less-economically developed or least-economically developed or less-affluent or least-affluent or deprived or poor) adj (country or countries or nation or nations or region or regions or economy or economies)).mp. |
| 29 | ((developing or underdeveloped or under-developed or less-developed or least-developed) adj (population* or world)).mp. |
| 30 | (third-world* or thirdworld* or 3rd-world* or lmic or lmics or lami countr* or lalmi countr* or transitional countr*).mp. |
| 31 | (low* adj (gdp or gnp or gross domestic or gross national)).mp. |
| 32 | ((underserved or under served) adj (countr* or nation? or population*)).mp. |
| 33 | 25 or 27 or 28 or 29 or 30 or 31 or 32 |
| 34 | 20 and 33 |
| 35 | (Mumbai or Delhi or Bangalore or Hyderabad or Ahmedabad or Chennai or Kolkata or Surat or Pune or Jaipur or Lucknow or Kanpur or Nagpur or Indore or Thane or Bhopal or Visakhapatnam or Pimpri Chinchwad or Patna or Vadodara or Ghaziabad or Ludhiana or Agra or Nashik or Faridabad or Meerut or Rajkot or Kalyan Dombivli or Vasai Virar or Varanasi or Srinagar or Aurangabad or Dhanbad or Amritsar or Navi Mumbai or Allahabad or Howrah or Ranchi or Gwalior or Jabalpur or Coimbatore or Vijayawada or Jodhpur or Madurai or Raipur or Kota or Chandigarh or Guwahati or Solapur or Hubli Dharwad or Tiruchirappalli or Bareilly or Mysore or Tiruppur or Gurgaon or Aligarh or Jalandhar or Bhubaneswar or Salem or Mira Bhayandar or Warangal or Jalgaon or Guntur or Bhiwandi or Saharanpur or Gorakhpur or Bikaner or Amravati or Noida or Jamshedpur or Bhilai or Cuttack or Firozabad or Kochi or Nellore or Bhavnagar or Dehradun or Durgapur or Asansol or Rourkela or Nanded or Kolhapur or Ajmer or Akola or Gulbarga or Jamnagar or Ujjain or Loni or Siliguri or Jhansi or Ulhasnagar or Jammu or Sangli Miraj or Kupwad or Mangalore or Erode or Belgaum or Ambattur or Tirunelveli or Malegaon or Gaya or Thiruvananthapuram or Udaipur or Maheshtala or Davanagere or Kozhikode or Kurnool or Rajpur Sonarpur or Rajahmundry or Bokaro or South Dumdum or Bellary or Patiala or Gopalpur or Agartala or Bhagalpur or Muzaffarnagar or Bhatpara or Panihati or Latur or Dhule or Tirupati or Rohtak or Korba or Bhilwara or Berhampur or Muzaffarpur or Ahmednagar or Mathura or Kollam or Avadi or Kadapa or Kamarhati or Sambalpur or Bilaspur or Shahjahanpur or Satara or Bijapur or Kakinada or Rampur or Shimoga or Chandrapur or Junagadh or Thrissur or Alwar or Bardhaman or Kulti or Nizamabad or Parbhani or Tumkur or Khammam or Ozhukarai or Bihar Sharif or Panipat or Darbhanga or Bally or Aizawl or Dewas or Ichalkaranji or Karnal or Bathinda or Jalna or Eluru or Barasat or Kirari Suleman Nagar or Purnia or Satna or Mau or Sonipat or Farrukhabad or Sagar or Durg or Imphal or Ratlam or Hapur or Arrah or Anantapur or Karimnagar or Etawah or Ambarnath or North Dumdum or Bharatpur or Begusarai or New Delhi or Gandhidham or Baranagar or Tiruvottiyur or Pondicherry or Sikar or Thoothukudi or Rewa or Mirzapur or Raichur or Pali or Ramagundam or Haridwar or Vijayanagaram or Katihar or Nagercoil or Sri Ganganagar or Karawal Nagar or Mango or Thanjavur or Bulandshahr or Uluberia or Katni or Sambhal or Singrauli or Nadiad or Secunderabad or Naihati or Yamunanagar or Bidhannagar or Pallavaram or Bidar or Munger or Panchkula or Burhanpur or Raurkela or Kharagpur or Dindigul or Gandhinagar or Hospet or Nangloi Jat or Malda or Ongole or Deoghar or Chapra or Haldia or Khandwa or Nandyal or Morena or Amroha or Anand or Bhind or Bhalswa Jahangir Pur or Madhyamgram or Bhiwani or Berhampore or Ambala or Morbi or Fatehpur or Raebareli or Mahaboobnagar or Chittoor or Bhusawal or Orai or Bahraich or Vellore or Mehsana or Raiganj or Sirsa or Danapur or Serampore or Sultan Pur Majra or Guna or Jaunpur or Panvel or Shivpuri or Surendranagar or Dudhrej or Unnao or Chinsurah or Alappuzha or Kottayam or Machilipatnam or Shimla or Adoni or Udupi or Tenali or Proddatur or Saharsa or Hindupur or Sasaram or Hajipur or Bhimavaram or Kumbakonam or Dehri or Madanapalle or Siwan or Bettiah or Guntakal or Srikakulam or Motihari or Dharmavaram or Gudivada or Phagwara or Narasaraopet or Suryapet or Miryalaguda or Tadipatri or Karaikudi or Kishanganj or Jamalpur or Ballia or Kavali or Tadepalligudem or Amaravati or Buxar or Jehanabad or Aurangabad or Gangtok).mp. |
| 36 | (Sabaragamuwa or Uva province or Kandy or Trincomalee or Anuradhapura or Jaffna or Kurunegala or Ratnapura or Galle or Badulla or Colombo or Ampara or Batticaloa or Gampaha or Hambantota or Kalutara or Kegalle or Kilinochchi or Mannar or Matale or Matara or Monaragala or Mullaitivu or Nuwara Eliya or Polonnaruwa or Puttalam or Vavuniya or Dehiwala or Moratuwa or Sri Jayawardenapura or Kotte or Negombo or Kalmunai or Vavuniya or Katunayake or Dambulla or Kolonnawa or Anuradhapura).mp. |
| 37 | (Dhaka or Chittagongor Rajshahi or Khulna or Sylhet or Mymensingh or Barisal or Rangpur or Comilla or Narayanganj or Gazipur or Bogra or Kushtia or Jessore or Cox's Bazar or Manikganj or Brahmanbaria or Dinajpur or Nawabganj or Tangail or Sirajganj or Chandpur or Feni or Jamalpur or Naogaon or Narsingdi or Pabna or Maijdee or Faridpur or Tongi or Rangpur or Savar or Siddhirganj or Jessore).mp. |
| 38 | (Kathmandu or Pokhara or Lalitpur or Biratnagar or Birganj or Dharan or Bharatpur or Bhim Dutta or Butwal or Hetauda or Bhaktapur or Janakpur or Dhangadhi or Itahari or Triyuga or Siddharthanagar or Nepalganj or Madhyapur Thimi or Mechinagar or Ghorahi or Lekhnath or Kirtipur or Birendranagar or Gulariya or Tikapur or Ratnanagar or Tulsipur or kalaiya or Kamalamai or Damak or Gorkha or Rajbiraj or Kapilvastu or Byas or Lahan or Putalibazar or Panauti or Gaur or Dipayal-Silgadhi or Inaruwa or Siraha or Ramgram or Jaleswar or Baglung or Tansen or Khandbari or Bhimeshwar or Dhankuta or Bidur or Waling or Narayan or Malangwa or Bhadrapur or Amaragadhi or Dasharathchand or Ilam or Banepa or Dhulikhel or Bagmati or Gandaki or Karnali or Sudurpaschim or Patan or Birgunj or Mahendranagar).mp. |
| 39 | (Karachi or Lahore or Faisalabad or Hyderabad or Rawalpindi or Gujranwala or Peshawar or Multan or Islamabad or Quetta or Sargodha or Bahawalpur or Sialkot or Sukkur or Larkana or Sheikhupura or Rahim Yar Khan or Jhang or Dera Ghazi Khan or Gujrat or Sahiwal or Wah Cantonment or Mardan or Kasur or Okara or Mingora or Nawabshah or Chiniot or Kotri or Kamoke or Hafizabad or Sadiqabad or Mirpur Khas or Burewala or Kohat or Khanewal or Dera Ismail Khan or Turbat or Muzaffargarh or Abbotabad or Mandi Bahauddin or Shikarpur or Jacobabad or Jhelum or Khanpur or Khairpur or Khuzdar or Pakpattan or Daska or Gojra or Dadu or Muridke or Bahawalnagar or Samundri or Tando Allahyar or Tando Adam or Jaranwala or Chishtian or Attock or Vehari or Kot Abdul Malik or Ferozwala or Gwadar ir Chakwal or Gujranwala Cantonment or Kamalia or Umerkot or Ahmedpur East or Kot Addu or Wazirabad or Mansehra or Layyah or Swabi or Chaman or Taxila or Nowshera or Khushab or Shahdadkot or Mianwali or Kabal or Lodhran or Hasilpur or Charsadda or Bhakkar or Badin or Arif Wala or Ghotki or Sambrial or Jatoi or Haroonabad or Daharki or Narowal or Tando Muhammad Khan or Kamber Ali Khan or Mirpur Mathelo or Kandhkot or Bhalwal or Azad Jammu or Kashmir or Balochistan or Gilgit Baltistan or Khyber Pakhtunkhwa or Punjab or Sindh).mp. |
| 40 | (Jakarta or Surabaya or Bekasi or Bandung or Medan or Depok or Tangerang or Palembang or Semarang or Makassar or South Tangerang or Batam or Bandar Lampung or Bogor or Pekanbaru or Padang or Malang or Samarinda or Denpasar or Tasikmalaya or Serang or Balikpapan or Pontianak or Banjarmasin or Jambi or Cimahi or Surakarta or Manado or Kupang or Cilegon or Mataram or Jayapura or Bengkulu or Yogyakarta or Palu or Ambon or Sukabumi or Kendari or Cirebon or Dumai or Pekalongan or Palangka Raya or Binjai or Kediri or Sorong or Tegal or Pematangsiantar or Banjarbaru or Banda Aceh or Tarakan or Probolinggo or Singkawang or Lubuklinggau or Tanjungpinang or Bitung or Padang Sidempuan or Pangkalpinang or Batu or Pasuruan or Ternate or Banjar or Gorontalo or Madiun or Prabumulih or Salatiga or Lhokseumawe or Langsa or Palopo or Bontang or Tanjungbalai or Tebing Tinggi or Bau Bau or Bima or Parepare or Blitar or Pagar Alam or Payakumbuh or Gunungsitoli or Mojokerto or Kotamobagu or Magelang or Bukittinggi or Tidore or Tomohon or Sungai Penuh or Pariaman or Subulussalam or Sibolga or Tual or Solok or Sawahlunto or Padang Panjang or Sabang or Java or Sumatra or Sulawesi or Kalimantan or Lesser Sunda Islands or Western New Guinea or Maluku Islands).mp. |
| 41 | (Kuala Lumpur or Seberang Perai or Subang Jaya or George Town or Ipoh or Petaling Jaya or Shah Alam or Iskandar Puteri or Johor Bahru or Malacca or Kota Kinabalu or Kuantan or Alor Setar or Kuala Terengganu or Kuching or Seremban or Miri or Pasir Gudang or Penang or Selangor or Perak or Johor or Sabah or Pahang or Kedah or Terengganu or Sarawak or Negeri Sembilan).mp. |
| 42 | (Bangkok or Nonthaburi or Pak Kret or Hat Yai or Chaophraya Surasak or Surat Thani or Nakhon Ratchasima or Chiang Mai or Udon Thani or Pattaya or Khon Kaen or Nakhon Si Thammarat or Laem Chabang or Rangsit or Nakhon Sawan or Phuket or Chiang Rai or Ubon Ratchathani or Nakhon Pathom or Ko Samui or Samut Sakhon or Phitsanulok or Rayong or Songkhla or Yala or Trang or Om Noi or Sakon Nakhon or Lampang or Samut Prakan or Phra Nakhon Si Ayutthaya or Mae Sot).mp. |
| 43 | (Ho Chi Minh City or Hanoi or Haiphong or Can Thu or Da Nang or Bien Hoa or Thu Duc or "Thuan An" or Hai Duong or Dong Nai or Thua Thien Hue or Binh Duong or Nha Trang or "Di An" or Buon Ma Thuot or Thanh Hoa or Vung Tau or Thai Nguyen or Vinh or Thu Dau Mot or "Ha Long" or Quy Nhon or Khanh Hoa or Dak Lak or Ba Ria Vung Tau or "Nghe An" or Quang Ninh or Binh Dinh).mp. |
| 44 | 35 or 36 or 37 or 38 or 39 or 40 or 41 or 42 or 43 |
| 45 | 20 and 44 |
| 46 | 26 or 34 or 45 |
| 47 | 33 or 44 |
| 48 | 5 and 8 and 47 |

*Note:* This search strategy was developed for Cochrane
