# Supplementary material for: The effectiveness of diabetes self-management education intervention on glycaemic control and cardiometabolic risk in adults with type 2 diabetes in low- and middle-income countries: A systematic review and meta-analysis
Source: PLoS One. 2024 Feb 2;19(2):e0297328. doi: 10.1371/journal.pone.0297328 (PMC10836683; doi:10.1371/journal.pone.0297328)
Supplement: S4 Table — (DOCX) [file pone.0297328.s004.docx]

**Table S4** Primary and secondary outcomes

| **Outcome measures** | **Outcome types** | **Measures** |
| --- | --- | --- |
| Primary | Clinical outcomes | HbA1c and FBG |
| Secondary | Cardiometabolic risk factors | BMI, WC, TC, LDL, HDL, TG, SBP and DBP |
|  | Diabetes self-management behaviours | Diabetes Knowledge and Self-efficacy |
|  | Psychosocial well-beign | HrQoL |

*HBA1c: glycosylated haemoglobin, FBG: fasting blood glucose, BMI: Body mass index, WC: Waist circumstance, LDL: Low-density lipoprotein, HDL: High-density lipoprotein, TC: Total* Cholesterol*, TG: Triglyceride, SBP: Systolic blood pressure, DBP: Diastolic blood pressure, HrQoL: Health-related quality of life*
