# Supplementary material for: The effectiveness of diabetes self-management education intervention on glycaemic control and cardiometabolic risk in adults with type 2 diabetes in low- and middle-income countries: A systematic review and meta-analysis
Source: PLoS One. 2024 Feb 2;19(2):e0297328. doi: 10.1371/journal.pone.0297328 (PMC10836683; doi:10.1371/journal.pone.0297328)
Supplement: S5 Table — (DOCX) [file pone.0297328.s005.docx]

**Table S5** Other characteristics (intervention description) of the included studies

| **Sl No** | **First author (year)** | **Study design** | **Intervention description** |
| --- | --- | --- | --- |
| 1 | Askari et al (2018) | Randomised clinical trial | Training in 8 sessions (2 sessions in a week); each session lasted for 70 min; question and answer, exercise, discussion, image and messages were sent to the patients each week. The session content was about diabetes, signs and symptoms, diet, food composition tables, partitioning, proper use of fruits, vegetables, and grains as sources of dietary fibre. |
| 2 | Azami et al (2018) | Randomised control trial | 12 weeks nurse-led intervention with booklet, couseling on self-management, movie clips, 4 weeks of the group intervention and each session lasted 120 minutes, focused on building knowledge, self-efficacy and skills, follow-up telephone calls (weekly). |
| 3 | Baviskar et al (2020) | Randomsied control trial | Patients in the counselling groups identified problem areas such as lack of diet control, physical activity, education about self care and diabetes provided by the investigator and social worker, groups interviewed and motivated to time-boundound goals, given dietary plan and exercised schedule was prepared, goals were reviewed and revised during follow up. |
| 4 | Chow et al (2016) | Non-clinical randomised controlled trial | The first session focused on medication indications, dosage, administration frequencies, side effects and side effect management, storage and disposal of the medications and the importance of medication adherence. The second session focused on T2DM pathophysiology, risk factor, types of diabetes, sign and symptoms, diabetes complications, meal planning, lifestyle modifications and self-care. |
| 5 | Debussche et al (2018) | Randomised control trial | 3 group sessions on cardiovascular risk management, food intake, exercise, and blood glucose and insulin management. |
| 6 | Didarloo et al (2016) | Randomised control trial | Instructional sessions (group discussion, brainstorming and question and response techniques. Especially, for promoting the self‑efficacy of diabetics, the educator utilized specific training approaches such as verbal persuasion, modeling, and performance accomplishments). Interactive discussion session: diabetes knowledge, attitutde toward behaviour, behavioural performance and health-realated QOL. |
| 7 | Ebrahimi et al (2016) | Double blind Randomised clinical trial | The training workshops were conducted among patients, their families and health workers. The content of the education was diet, exercise, medication, and foot care. Patients in the intervention group were divided into five groups of 10. According to patients’ needs, weekly regular meetings were held. |
| 8 | Essien et al (2017) | Individually-randomised controlled trial | 12 sessions on diet and nutrition, compliance with medications and the mechanism of medication action, exercise, foot and skin care, self-monitoring of glucose levels, smoking cessation and blood pressure and cholesterol monitoring. The initial six sessions were delivered by three doctors and the final six sessions were delivered by three nurses. |
| 9 | Gathu et al (2018) | Non-blinded randomised clinical trial | The education content included the American Association of Diabetes Educators (AADE) 7-core self-care behaviours; being active, nutrition, monitoring blood glucose and adherence to medication, among other topics. Indivisualised three (lasting one-hour) sessions in every six weeks and telephone reminder of the sessions and usual physician consultation in every 3 months. |
| 10 | Goldhaber-Fiebert et al (2003) | Randomised conrol trial | The Intervention group began the 12-week basic diabetes education and focused on portion control for weight reduction and use of healthier food. The intervention included 11 weekly nutrition classes (90 min each session) in centrally located community centers. |
| 11 | Goodarzi et al (2012) | Randomised conrol trial | 4 messages delivered weekly consisted of diet, exercise, diabetic medication, important of self-monitoring blood glucose levels. |
| 12 | Grillo et al (2016) | Single-center, parallel-group, randomised study | The course content included identification of modifiable risk factors for tye 2 diabetes mellitus, emphasizing diet and excersice, mechanism of action and side effects of glucose-lowering medications, an overview of chronic diabetes complications, and foot care. |
| 13 | Hosseini et al (2017) | Randomised control trial | Education was delivered by a general physician and a specialist in health education and promotion. Workshop sessions held for 4 weeks (2 hrs/week), in the final session patients were provided brochurs and necessary guidance. The content was educational intervention on self‑care behaviors among patients with diabetes, an application of PRECEDE model. |
| 14 | Huo et al (2017) | Randomised clinical trial | Six text messages per week across a 6-month time frame. Text messages about risk factor modification. Messages were theory driven and culturally tailored to provide educational and motivational information on glucose monitoring, blood pressure control, medication adherence, physical activity, and lifestyle. |
| 15 | Jain et al (2018) | Open-label randomised controlled trial | Intervention by the community health worker and visited the study participants at their homes every 6 weekly (at 6 weeks, 12 weeks, 18 weeks and end of study at 24 weeks). The intervention group was offered health education about the disease, its symptoms, its complications, various medications and lifestyle measures, and telephonic reminders. |
| 16 | Jayasuria et al (2015) | Randomised control trial | The dietary intervention focused on decreasing total energy intake, reducing the number of starchy foods (e.g. rice) replaced by vegetables/green leaves. The physical activity intervention targeted increasing culturally appropriate exercise during-household work (for women) and introducing brisk. The first four sessions within 6 weeks, following monthly (4 weekly) for 5 more visit. |
| 17 | Jiang et al (2019) | Multicentre randomised controlled trial | The components were diabetes‐related knowledge and diabetes self-management skills based on self‐efficacy theory. The intervention was given 4 weekly sessions for 1 month and then face to‐face/telephone meetings every 3 months. The delivery of four sessions was mainly completed by a trained education nurse, and a ten‐minute introduction of the programme was finished by a physician. |
| 18 | Ju et al (2018) | Cluster randomised control trial | Each group consisted of 10 –15 participants and one or two peer support leaders with diabetes and shared a variety of skills, including diabetes education, healthy meal planning, food preparation, blood glucose monitoring, medication management and physical activities. Peer leaders encouraged participants to communicate and share experience with each other. |
| 19 | Kong et al (2019) | Group Randomized Experimental Study | Booklets and face-to-face communication, continuous medical education; education was 9 sessions every month. Received the five components CCM-based intervention, awareness of the chronic disease management; self-management support included goals setting, planning, doing, checking and assessing. |
| 20 | Lamptey et al (2023) | Single-blind randomised parallel comparator controlled multi-centre trial | The intervention consisted of one session of structured DSME, delivered by two educators to groups of six to ten participants in one day, over 6 h. The delivery of the intervention was completed within 2 weeks of randomisation. The intervention was delivered by providers not directly involved in patient care. |
| 21 | Li et al (2016) | Randomized controlled trial | Participants in the intensive nutrition education group were arranged to receive intensive nutritional lectures about diabetes for 30 days. The contents were focused on knowledge of diabetes, diabetes medication, blood glucose monitoring, healthy diet, and healthy lifestyle. |
| 22 | Lou et al (2020) | Randomised control trial | First, this model integrates the CDC (centers for disease control and prevention), GHs (general hospitals), and local CHCs (community health service centers) into a single system to maximize the utility of each institute in clinic-based diabetes management. Second, patients with diabetes were invited to attend a health class to learn about their diabetes, and to join self-management activities in their local CHCs every month. |
| 23 | Mohammadi et al (2018) | A matched-pair design randomized controlled trial | Patients were educated about diabetes and its complications, self-care and self-efficacy behavior, physical activity, healthy diet, medication adherence and to self-monitor their blood glucose level. 8 sessions (2 hours per session) with an educational booklet. |
| 24 | Muchiri et al (2014) | Randomised control trial | Eight weekly sessions of group nutrition education sessions on the treatment of diabetes, dietary guidelines, meal planning, and meal preparation. |
| 25 | Myers et al (2017) | Cluster randomised control trial | All patients received nutrition education handouts, such as meal plans, exchange lists, diabetes instructions, or daily routines. Other nutrition intervention strategies reported included motivational interviewing, goal setting, and self-monitoring blood glucose equipment. |
| 26 | Mash et al (2014) | Pragmatic clustered randomized controlled trial | The intervention consisted of four 60-min sessions of group education that focused on understanding diabetes, living a healthy lifestyle, understanding the medication and avoiding complications. |
| 27 | Ojieabu et al (2017) | Randomised control trial | One educational session per month (total session 4) and counselled on the need for medication and treatment adherence such as clinic visits and lifestyle modifications including diet and exercise. |
| 28 | Ramadas et al (2018) | Multi-centre randomised control trial | Twelve dietary lesson plans in the intervention package, personalised according to the patients’ dietary stages of change (DSOC) and expected to improve dietary knowledge, attitude, and behavior (DKAB) and to assist to progress in their respective DSOC. |
| 29 | Ramli et al (2016) | Pragmatic cluster randomised controlled trial | 1. Family medicine led the team, provided clinical expertise as the key resource person  2. Medical assistant/nurse-delivered education and counselling to empower patients with self-management skills  3. The medical officer managed patients and review treatment goals  4. Pharmacist monitored adherence to medications  5. The dietician/nutritionist delivered dietary advice and discussed meal plan |
| 30 | Samtia et al (2013) | Randomized study | A five month's educational intervention (by five pharmacists) regarding knowledge and self-care activities of diabetes. |
| 31 | Sanaeinasab et al (2021) | Randomised controlled trial | Six face-to-face 90-minute sessions were held on a weekly basis using small groups and the content focused on the most important issues regarding diabetes self-care. |
| 32 | Salahshouri (2018) | Randomised control trial | In eight educational sessions (lasting one hour), internal specialists, dieticians, diabetes specialists, psychologists and religious experts will conduct psychological and nutrition assessments. |
| 33 | Tan et al (2011) | Single-blind randomised control trial | The first session, healthy eating, being active, medication adherence and self-monitoring of blood glucose; the second and third sessions on problem-solving skills related to hyperglycaemia, hypoglycaemia, sick day and emotional episodes. |
| 34 | Thanh et al (2021) | Randomized controlled single-center trial | Diet and balanced nutrition (month 1), how to maintain a suitable exercise program (month 2), and use of type 2 diabetes medication (month 3). Education given in the form of three 45-min sessions led by trained medical staff educators on diet, exercise, drug therapy and adherence. |
| 35 | Wattana et al (2007) | Randomised controlled trial | One diabetes education class (120 min); four small group discussions (90 min/group); two individual home visit sessions from the researcher (45 min), and a patient education manual. The health education followed institutional guidelines without a structured program during the waiting time for a follow-up visit at the diabetic clinic. |
| 36 | Whittemore et al (2020) | Randomised control trial | Seven interactive group-based educational sessions on diabetes self-management; behavioral support provided in all sessions by class leaders to assist in problem-solving barriers to change and to develop weekly goals (including follow-up phone calls every 2 week). Lastly, daily text/picture messages, sent daily for 6 months maintenance of behavior change. |
| 37 | Wichit et al (2016) | Randomised controlled trial | Three educational sessions at baseline, week 5 and week 9; provided diabetes information wordbook included self-help worksheets and daily diary to record all daily activities including newly learned healthcare practices. Diabetes complications, problem solving, meal planning, foot hygiene, and enhanced diabetes knowledge and competency were all featured in the program. |
| 38 | Yan et al (2014) | Randomised study | Group exercised under supervision, 45 min session (3–5 times/week). Supervised exercise of low intensity exercise (LEX) to vigorous intensity exercise (VEX) for 12 weeks. |
| 39 | Zhang et al (2018) | Randomised study | Developed specifically nine components: a low literacy color booklet, a motivational video, restricted diet with a plate, WeChat group and regular health lectures, targeted treatment, group medical visits, improvement of the uptake and maintenance of medication regimes, lifestyle interventions, and self-management educational interventions. These were accomplished through systematic health education in a group provided by health educators. |
| 40 | Zheng et al (2019) | Randomised controlled trial | Theory course: the intervention group was given 2-session diabetes self-management education besides the regular education programme such as diet guidance, exercise guidance, and knowledge of hypoglycaemia treatment, foot care, medication, and blood glucose monitoring. Practical course: nutrition guidance was applied by the dietitians after the first theory class and lasted for 40 minutes and individualized exercise guidance applied after the second theory class and lasted for 60 minutes. |
| 41 | Zhong et al (2015) | Randomised study | Twelve biweekly education meetings over 6 months to be co-led by peer leaders with CHSC staff involvement titrated to peer leaders’ needs. Meetings lasted 1.5 to 2 hours and covered a range of topics such as diet, physical activity, medications, foot care, stress management and depression, barriers to self-management, and obtaining resources and support from the community, family, friends, and the health system. |
| 42 | Al-Halaweh et al (2018) | Quasi-experimental study | The mobile clinic team provides comprehensive diabetes assessment and care to patients and families, including counseling with diabetologists, nurses, and nutritionists, to foster healthy lifestyle choices. The team provides direct services to diabetic patients, adjusts their treatment plan, and teaches the DCCM protocol to the local clinic team. |
| 43 | Pamungkas et al (2019) | Quasi-experimental research | The program comprised reflection on current behaviour,  assessment on DMSM practice and measuring the clinical outcomes, and sharing experience of diabetes management and problem on diabetes management; goal setting on how to perform regular physical activity, selecting healthy food; and small group discussion on preparing healthy food for diabetes patients, importance of physical activity and how to do an exercise, blood glucose monitoring as well as medication adherence. Three sessions of role-playing and case studies were conducted to build up the skill in DMSM practice. |
| 44 | Kumari et al (2018) | Quasi-experimental prospective trial | Experienced dietician, diabetes education, physical trainer, and diabetologists gave lifestyle modification counselling sessions in the local language. |
