# Supplementary material for: The effectiveness of diabetes self-management education intervention on glycaemic control and cardiometabolic risk in adults with type 2 diabetes in low- and middle-income countries: A systematic review and meta-analysis
Source: PLoS One. 2024 Feb 2;19(2):e0297328. doi: 10.1371/journal.pone.0297328 (PMC10836683; doi:10.1371/journal.pone.0297328)
Supplement: S6 Table — (DOCX) [file pone.0297328.s006.docx]

**Table S6** Risk of bias summary for quasi-experimental studies

| **Authors** | **Q1** | **Q2** | **Q3** | **Q4** | **Q5** | **Q6** | **Q7** | **Q8** | **Q9** | **Overall Appraisal*** | **Comments _reason for exclusion** |
| --- | --- | --- | --- | --- | --- | --- | --- | --- | --- | --- | --- |
| Al-Halaweh et al (2019) | Yes | Yes | Yes | Yes | Yes | Yes | Yes | Yes | Yes | Included | N/A |
| Pamungkas et al (2020) | Yes | Yes | Yes | Yes | Yes | Yes | Yes | Yes | Yes | Included | N/A |
| Kumari et al (2018) | Yes | Yes | Yes | Yes | Yes | Yes | Yes | Yes | Yes | Included | N/A |

*Overall appraisal: review authors’ judgments about each risk of bias item for each included study

Q1: Is it clear in the study what is the ‘cause’ and what is the ‘effect’ (i.e. there is no confusion about which variable comes first)?

Q2: Were the participants included in any comparisons similar?

Q3: Were the participants included in any comparisons receiving similar treatment/care, other than the exposure or intervention of interest?

Q4: Was there a control group?

Q5: Were there multiple measurements of the outcome both pre and post the intervention/exposure?

Q6: Was follow up complete and if not, were differences between groups in terms of their follow up adequately described and analyzed?

Q7: Were the outcomes of participants included in any comparisons measured in the same way?

Q8: Were outcomes measured in a reliable way?

Q9: Was appropriate statistical analysis used?
