# Supplementary material for: The effectiveness of diabetes self-management education intervention on glycaemic control and cardiometabolic risk in adults with type 2 diabetes in low- and middle-income countries: A systematic review and meta-analysis
Source: PLoS One. 2024 Feb 2;19(2):e0297328. doi: 10.1371/journal.pone.0297328 (PMC10836683; doi:10.1371/journal.pone.0297328)
Supplement: S7 Table — (DOCX) [file pone.0297328.s007.docx]

| **Table S7** GRADEpro level of quality evidence assessment  Summary of findings: | | | | | | |
| --- | --- | --- | --- | --- | --- | --- |
| Education intervention compared with usual care for people with type 2 diabetes mellitus | | | | | | |
| **Patient or population:** people with type 2 diabetes mellitus  **Setting:**  Intervention: education interventions;  Comparison: usual care | | | | | | |
| Outcome № of participants (studies) | Relative effect (95% CI) | **Anticipated absolute effects (95% CI)** | | | Certainty | What happens |
|  |  |  |  | **Difference** |  |  |
| Glycosylated Haemoglobin (HbA1c) follow-up: range 4 weeks to 348 weeks № of participants: 10500 (39 RCTs) | - |  | - | MD **0.64 higher** (0.45 higher to 0.83 higher) | ⨁⨁◯◯ Low | a. Randomisation process, deviations from the intended interventions, missing outcome data, measurement of the outcome and selection of the reported result;  b. Heterogeneity (I^2^>75%) |
| Body mass index (BMI) follow-up: range 4 weeks to 348 weeks № of participants: 7193 (23 RCTs) | - |  | - | MD **0.6 higher** (0.32 higher to 0.88 higher) | ⨁⨁◯◯ Low | a. Bias was assessed to be at 'high risk in the trial;  b. Inconsistency was high in the trial. |
| Low density lipoprotein (LDL) follow-up: range 4 weeks to 348 weeks № of participants: 5803 (18 RCTs) | - |  | - | MD **4.33 higher** (2.33 higher to 6.65 higher) | ⨁◯◯◯ Very low | a. Randomisation process, deviations from the intended interventions, missing outcome data, measurement of the outcome and selection of the reported result;  b. Heterogeneity (^I2^>75%); c. Wide confidence interval |
| High density lipoprotein (HDL) follow-up: range 4 weeks to 348 weeks № of participants: 5301 (17 RCTs) | - |  | - | MD **1.35 fewer** (2.6 fewer to 0.02 more) | ⨁⨁⨁◯ Moderate | a. Heterogeneity (I^2^>75%) b. Wide confidence interval |
| Total cholesterol (TC) follow-up: range 4 weeks to 348 weeks № of participants: 6763 (17 RCTs) | - |  | - | MD **4.5 higher** (0.32 higher to 8.68 higher) | ⨁◯◯◯ Very low | a. Randomisation process, deviations from the intended interventions, missing outcome data, measurement of the outcome and selection of the reported result;  b. Heterogeneity (I^2^>75%); c. Wide confidence interval |
| Triglyceride (TG) follow-up: range 4 weeks to 348 weeks № of participants: 6763 (12 RCTs) | - |  | - | MD **14.8 more** (8.18 more to 21.43 more) | ⨁◯◯◯ Very low | a. Randomisation process, deviations from the intended interventions, missing outcome data, measurement of the outcome, and selection of the reported result  b. Heterogeneity (I^2^>75%); c. Wide confidence interval |
| Waist circumference (WC) follow-up: range 12 weeks to 104 weeks № of participants: 4173 (10 RCTs) | - |  | - | MD **0.37 more** (1.89 fewer to 2.63 more) | ⨁⨁⨁◯ Moderate | a. Heterogeneity (I2>75%) |
| Fasting blood glucose (FBG) follow-up: range 4 weeks to 104 weeks № of participants: 5370 (19 RCTs) | - |  | - | MD **0.74 higher** (0.57 higher to 0.91 higher) | ⨁⨁◯◯ Low | a. Randomisation process, deviations from the intended interventions, missing outcome data, measurement of the outcome, and selection of the reported result;  b. Heterogeneity (I^2^>75%) |
| ***The risk in the intervention group** (and its 95% confidence interval) is based on the assumed risk in the comparison group and the **relative effect** of the intervention (and its 95% CI).  **CI:** confidence interval; **MD:** mean difference | | | | | | |
| **GRADE Working Group grades of evidence** **High certainty:** we are very confident that the true effect lies close to that of the estimate of the effect. **Moderate certainty:** we are moderately confident in the effect estimate: the true effect is likely to be close to the estimate of the effect, but there is a possibility that it is substantially different. **Low certainty:** our confidence in the effect estimate is limited: the true effect may be substantially different from the estimate of the effect. **Very low certainty:** we have very little confidence in the effect estimate: the true effect is likely to be substantially different from the estimate of effect. | | | | | | |
