# Supplementary figures and images for: The effectiveness of diabetes self-management education intervention on glycaemic control and cardiometabolic risk in adults with type 2 diabetes in low- and middle-income countries: A systematic review and meta-analysis
Source: PLoS One. 2024 Feb 2;19(2):e0297328. doi: 10.1371/journal.pone.0297328 (PMC10836683; doi:10.1371/journal.pone.0297328)

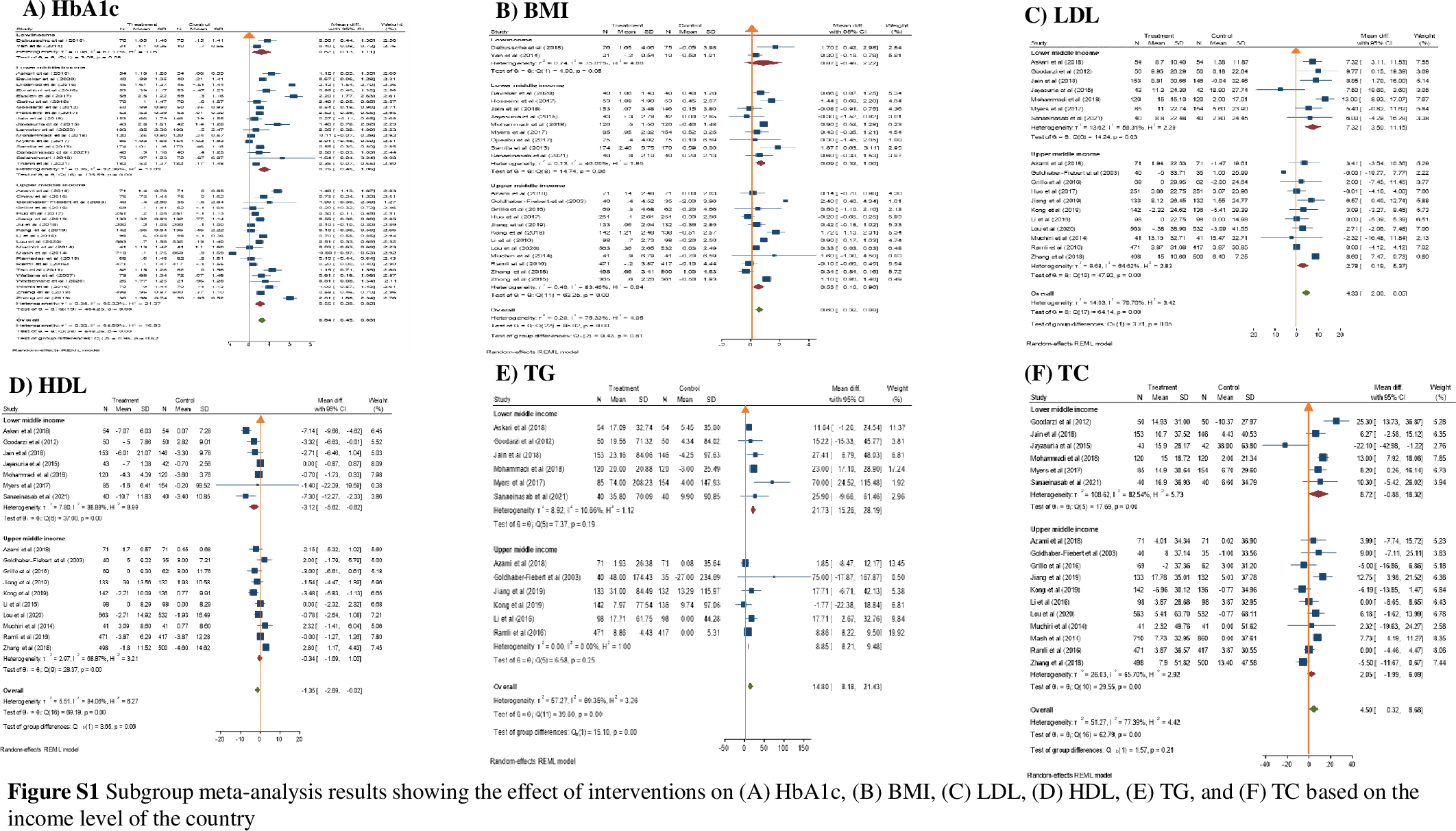

Supplement: S1 Fig — Subgroup meta-analysis results showing the effect of interventions on (A) HbA1c, (B) BMI, (C) LDL, (D) HDL, (E) TG, and (F) TC based on the income level of the country. (TIF) [file pone.0297328.s008.tif]

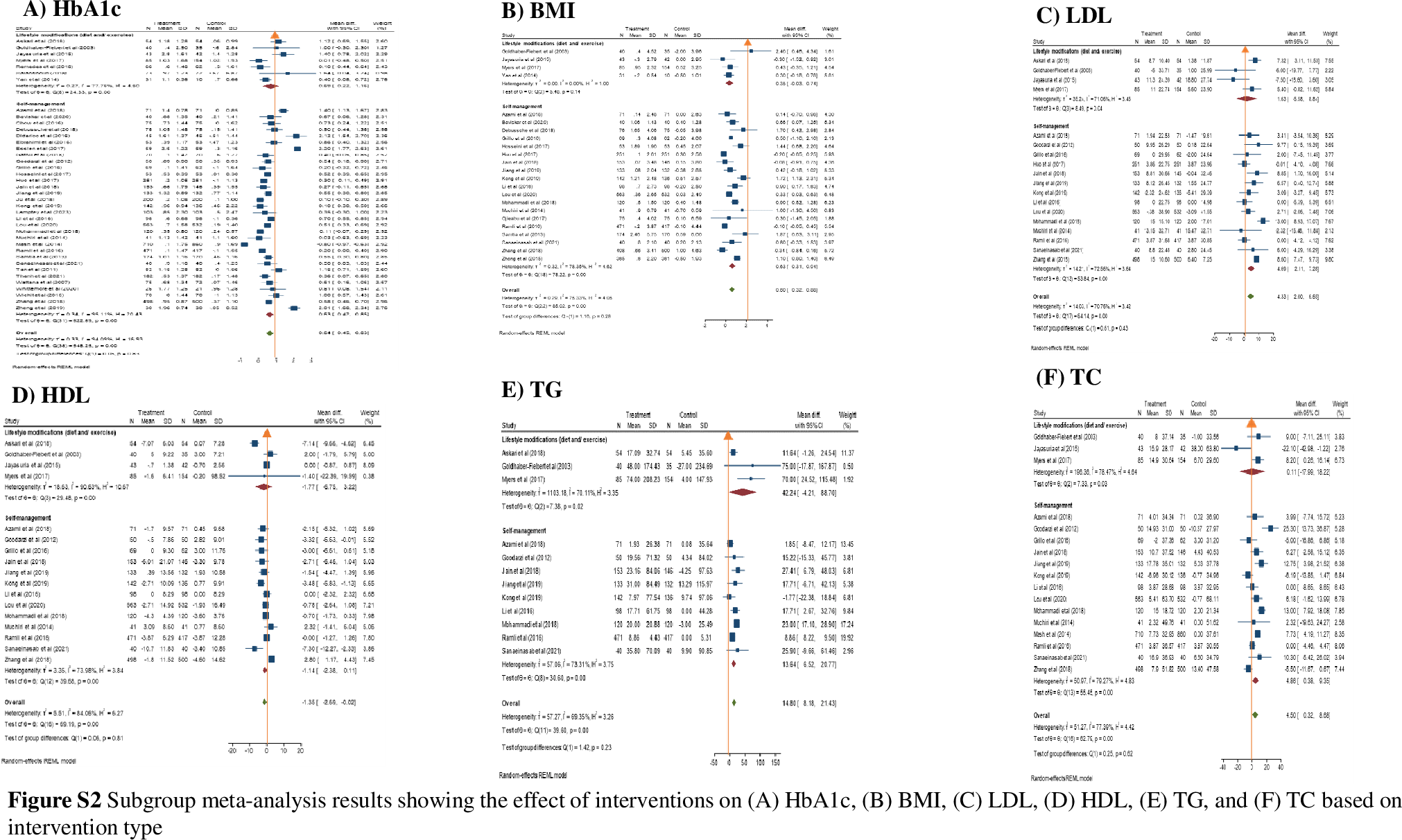

Supplement: S2 Fig — Subgroup meta-analysis results showing the effect of interventions on (A) HbA1c, (B) BMI, (C) LDL, (D) HDL, (E) TG, and (F) TC based on intervention type. (TIF) [file pone.0297328.s009.tif]

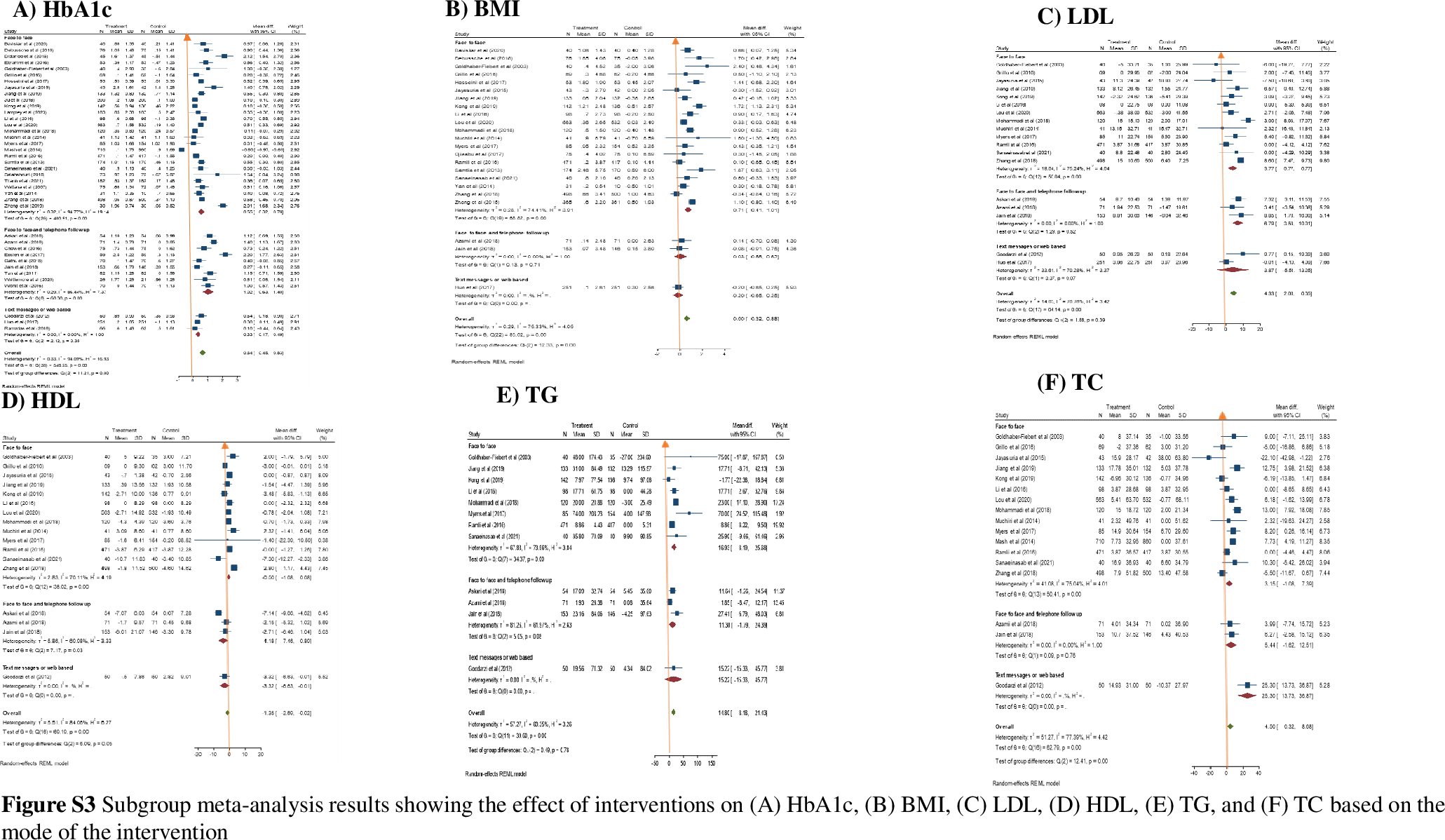

Supplement: S3 Fig — Subgroup meta-analysis results showing the effect of interventions on (A) HbA1c, (B) BMI, (C) LDL, (D) HDL, (E) TG, and (F) TC based on the mode of delivery of intervention. (TIF) [file pone.0297328.s010.tif]

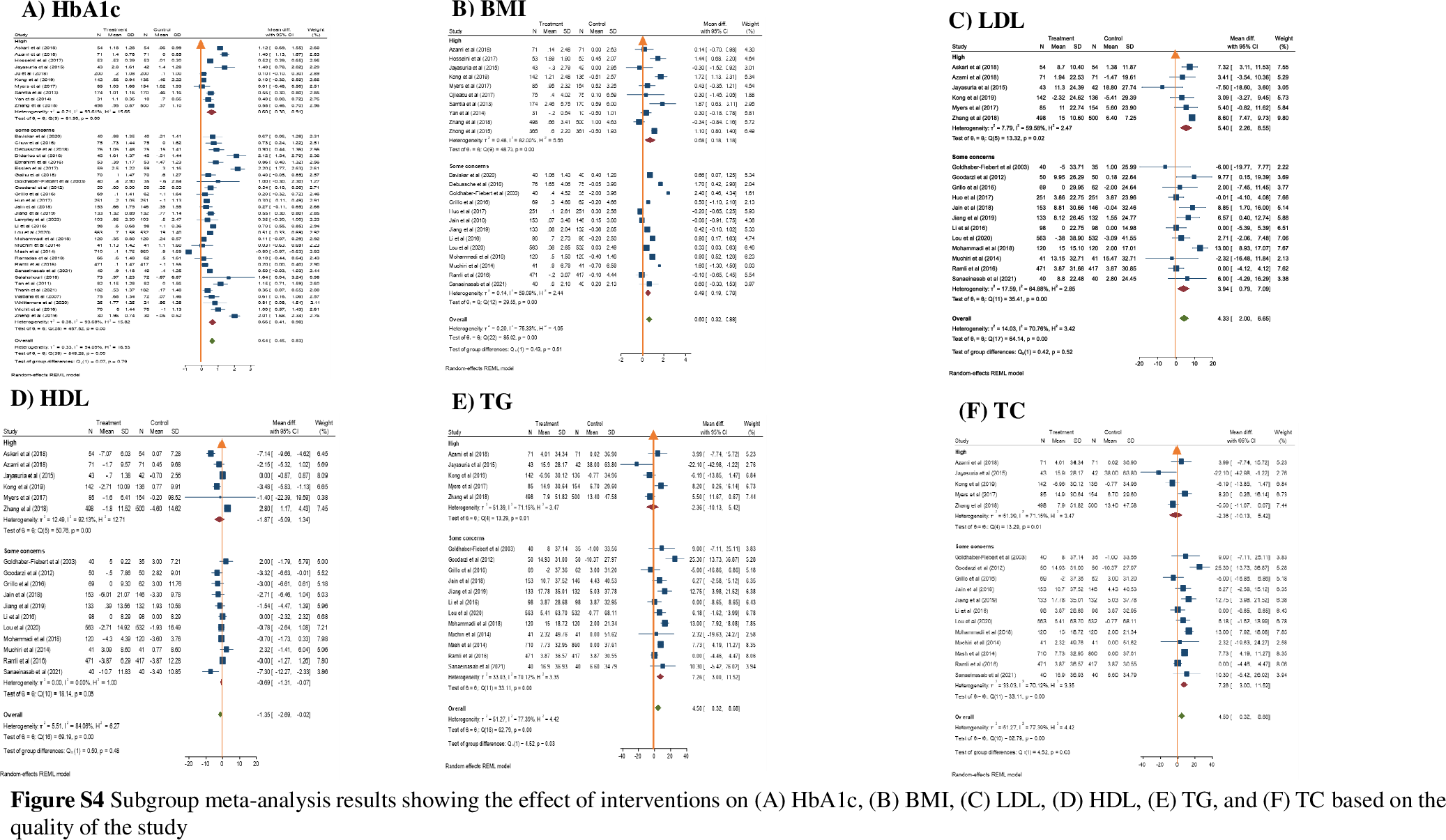

Supplement: S4 Fig — Subgroup meta-analysis results showing the effect of interventions on (A) HbA1c, (B) BMI, (C) LDL, (D) HDL, (E) TG, and (F) TC based on the quality of study. (TIF) [file pone.0297328.s011.tif]

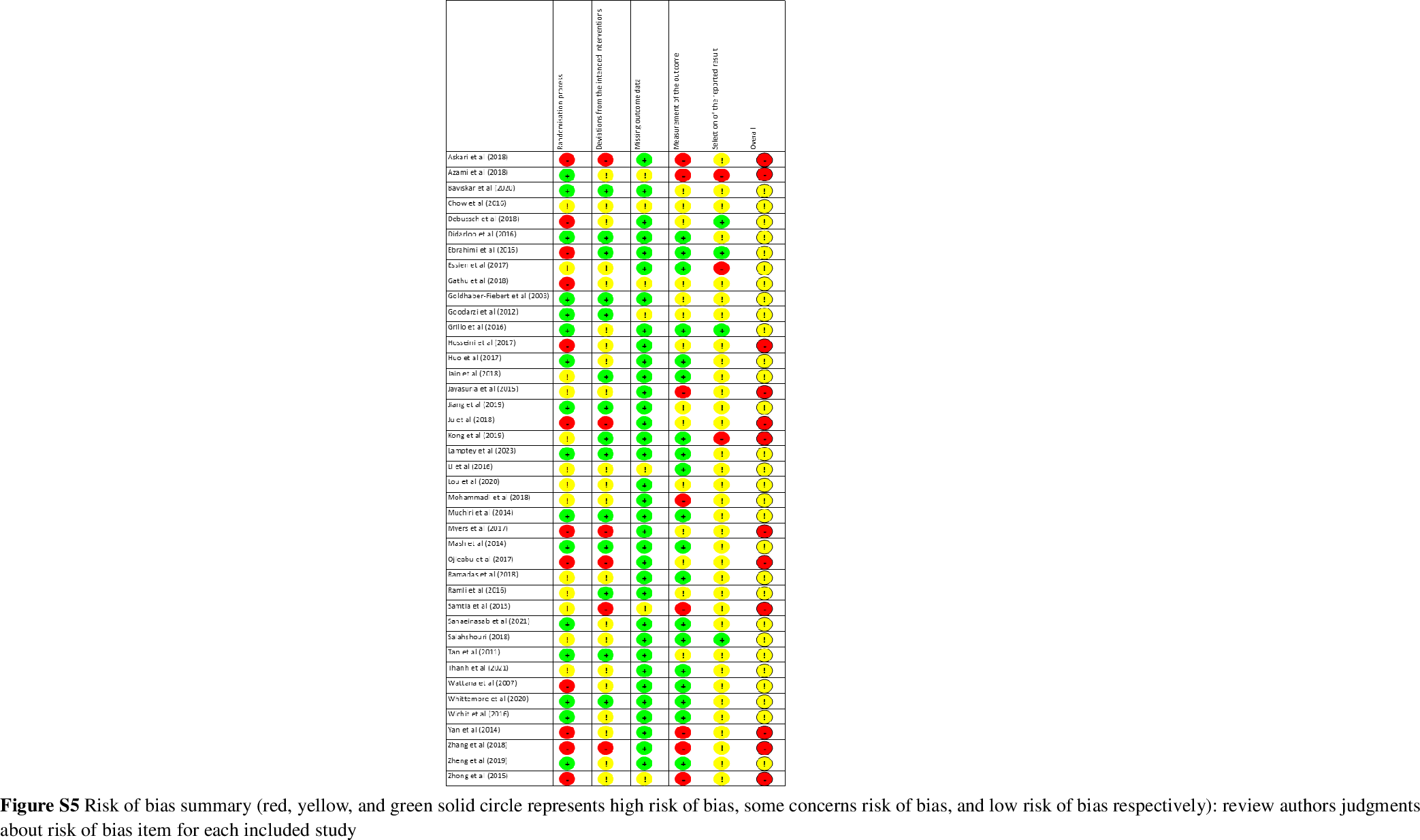

Supplement: S5 Fig — (TIF) [file pone.0297328.s012.tif]
